# Supplementary material for: Delayed processing of blood samples impairs the accuracy of mRNA-based biomarkers
Source: Sci Rep. 2022 May 17;12:8196. doi: 10.1038/s41598-022-12178-5 (PMC9113984; doi:10.1038/s41598-022-12178-5)
Supplement: Supplementary file 1 — Supplementary Information 1. [file 41598_2022_12178_MOESM1_ESM.pdf]

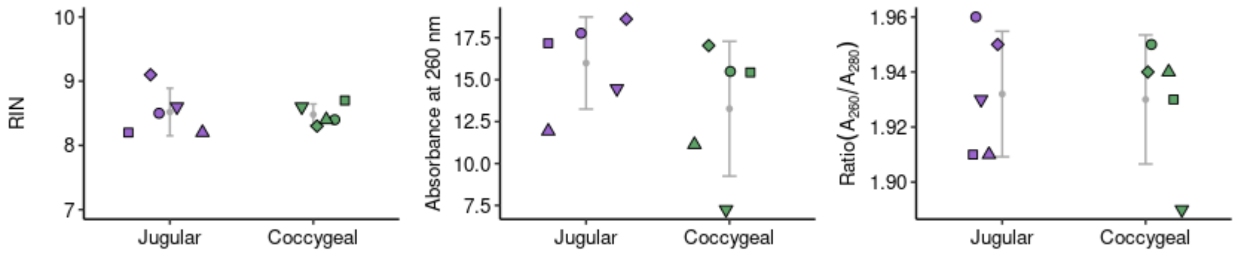

Supplementary Figure S1. Ribonucleic acid quality and abundance parameters from samples collected from the jugular and coccygeal veins and processed within one hour of sampling. Animals are indicated by shapes across charts. Center gray dot indicates average and vertical gray bars indicate standard deviation.

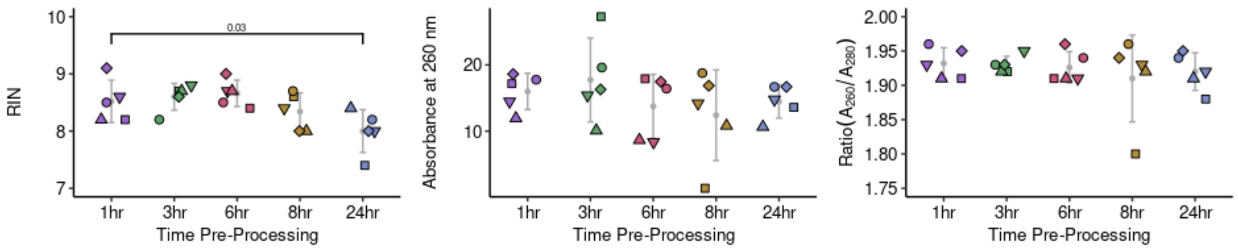

Supplementary Fig. S2. Ribonucleic acid quality and abundance parameters from samples collected from the jugular vein and processed after refrigeration (4°C) for multiple windows of time (x-axis). Animals are indicated by shapes across charts. Center gray dot indicates average and vertical gray bars indicate standard deviation.

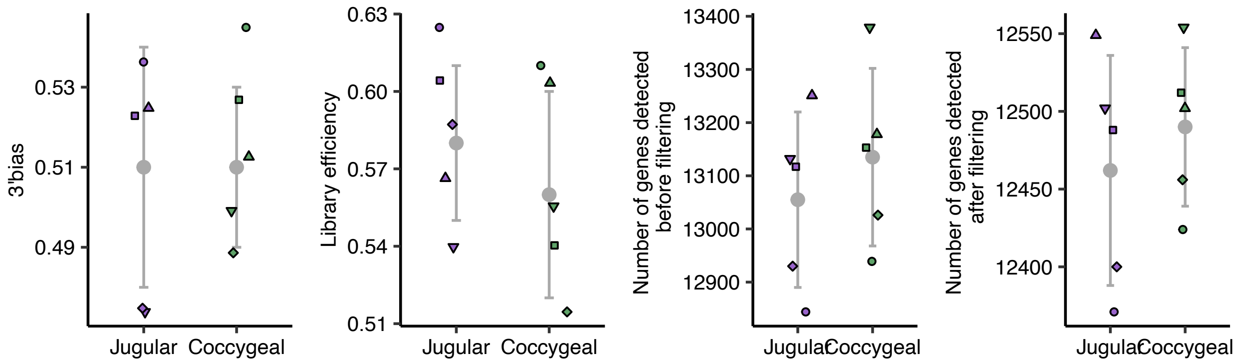

Supplementary Fig. S3. Parameters of library quality from samples collected from the jugular and coccygeal veins and processed within one hour of sampling. Animals are indicated by shapes across charts.

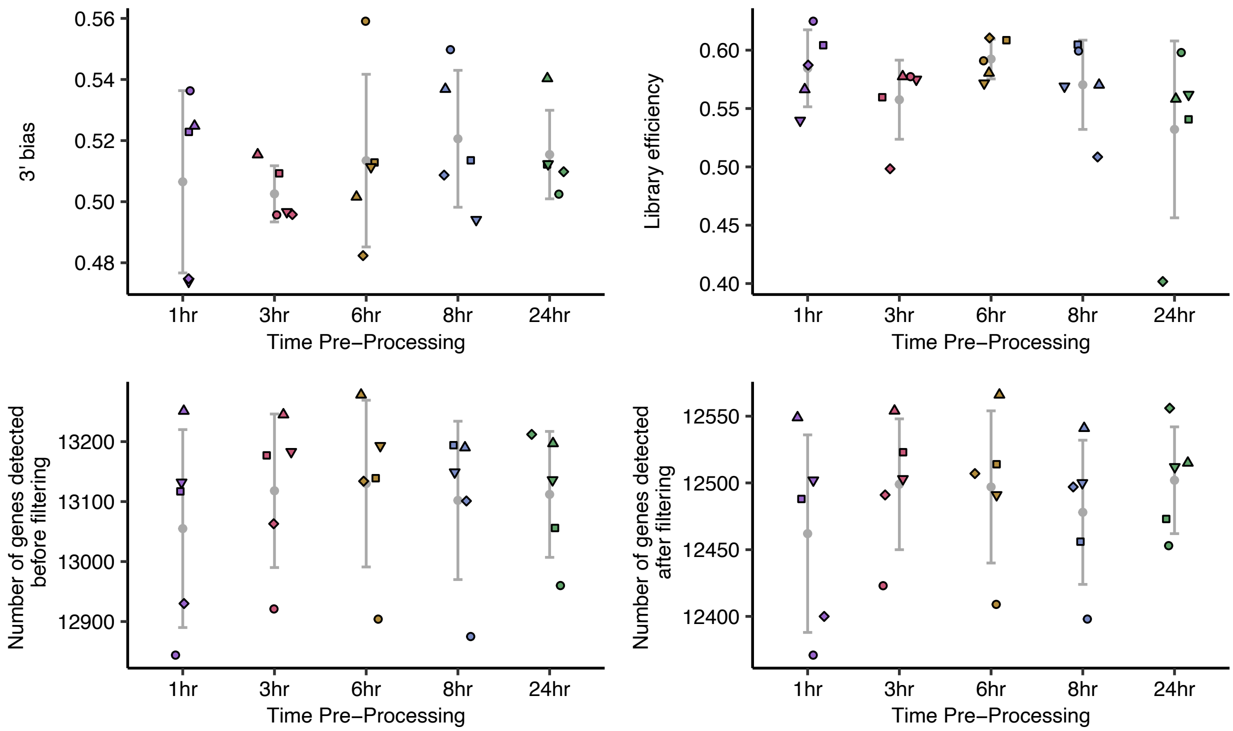

Supplementary Fig. S4. Parameters of library quality from samples collected from the jugular and processed after refrigeration (4°C) for multiple windows of time (x-axis). Animals are indicated by shapes across charts.

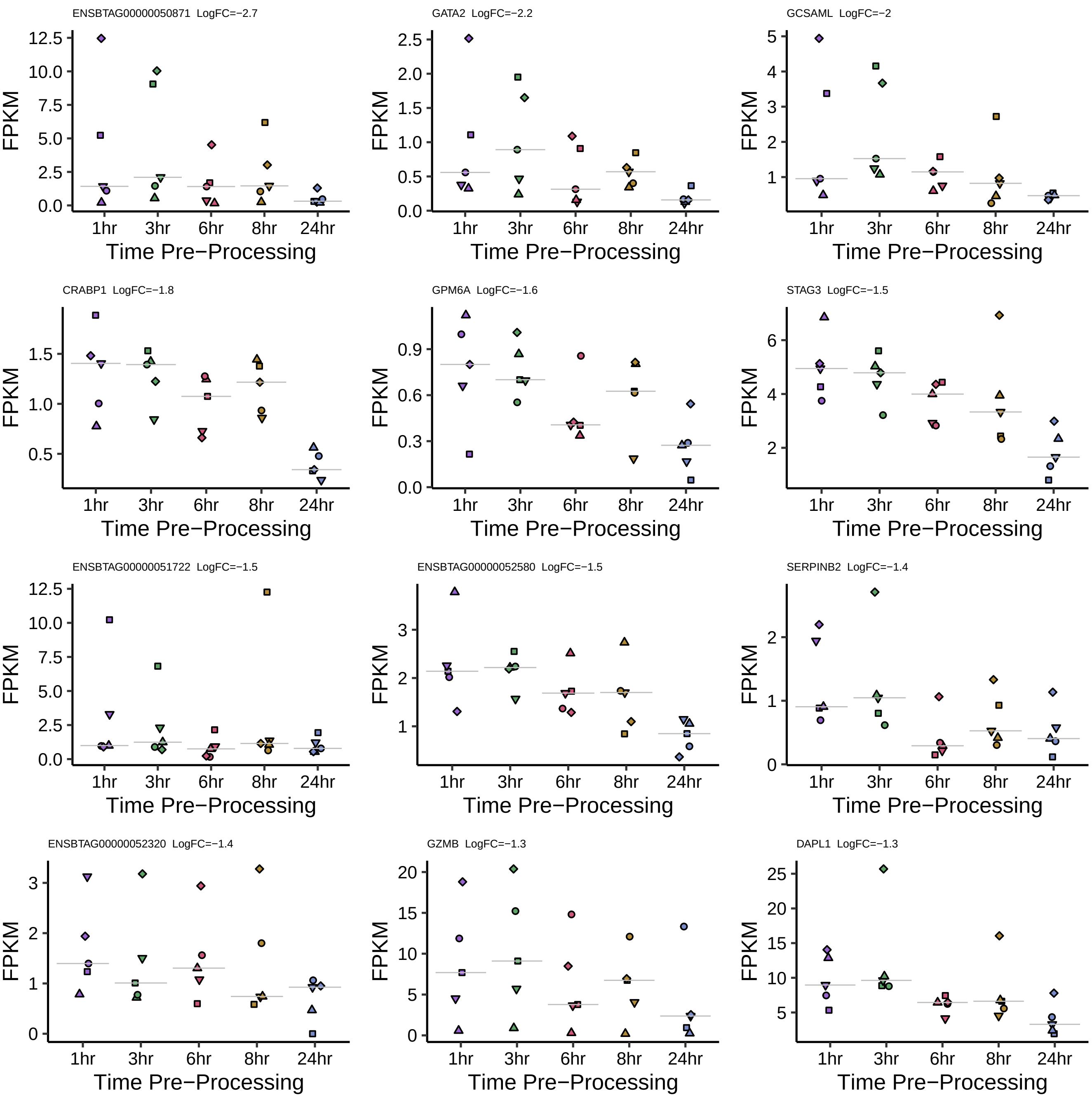
ENSBTAG00000050871 LogFC=−2.7
GATA2 LogFC=−2.2
GCSAML LogFC=−2
CRABP1 LogFC=−1.8
GPM6A LogFC=−1.6
STAG3 LogFC=−1.5
ENSBTAG00000051722 LogFC=−1.5
ENSBTAG00000052580 LogFC=−1.5
SERPINB2 LogFC=−1.4
ENSBTAG00000052320 LogFC=−1.4
GZMB LogFC=−1.3
DAPL1 LogFC=−1.3
FPKM
1hr
3hr
6hr
8hr
24hr
Time Pre−Processing

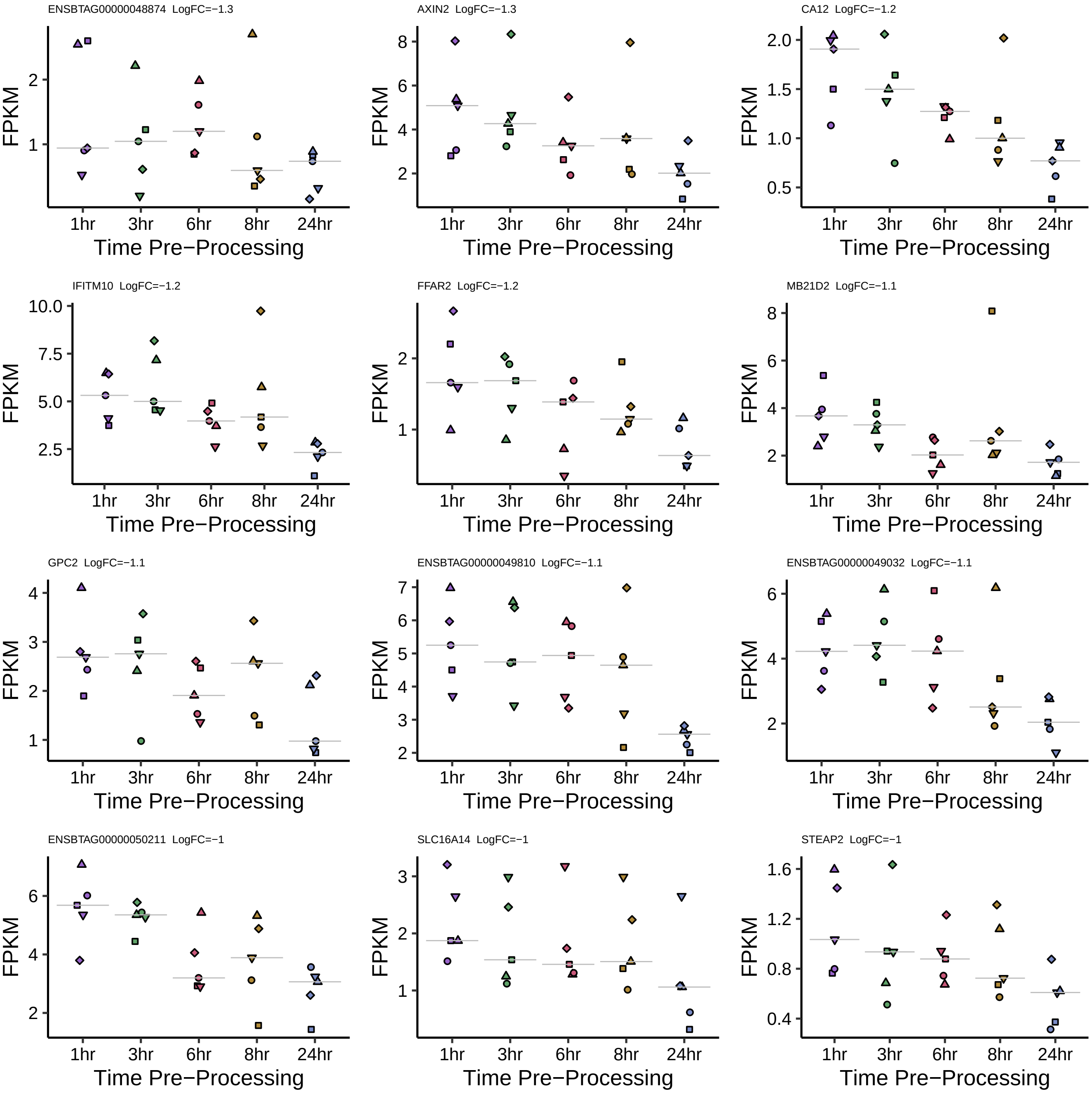

ENSBTAG00000048874 LogFC=−1.3
AXIN2 LogFC=−1.3
CA12 LogFC=−1.2
IFITM10 LogFC=−1.2
FFAR2 LogFC=−1.2
MB21D2 LogFC=−1.1
GPC2 LogFC=−1.1
ENSBTAG00000049810 LogFC=−1.1
ENSBTAG00000049032 LogFC=−1.1
ENSBTAG00000050211 LogFC=−1
SLC16A14 LogFC=−1
STEAP2 LogFC=−1
FPKM
1hr 3hr 6hr 8hr 24hr
Time Pre−Processing

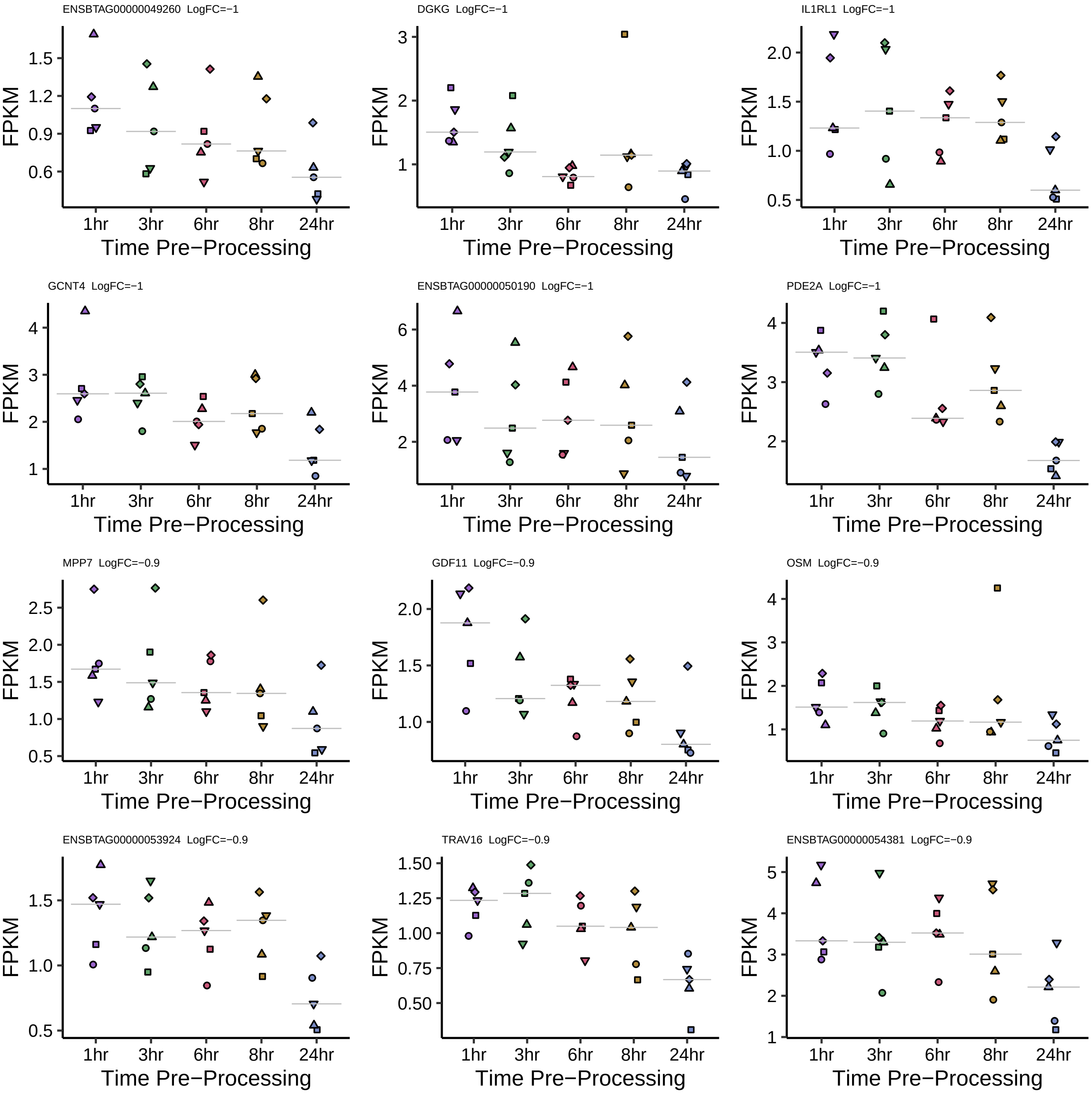
ENSBTAG00000049260 LogFC=−1
DGKG LogFC=−1
IL1RL1 LogFC=−1
GCNT4 LogFC=−1
ENSBTAG00000050190 LogFC=−1
PDE2A LogFC=−1
MPP7 LogFC=−0.9
GDF11 LogFC=−0.9
OSM LogFC=−0.9
ENSBTAG00000053924 LogFC=−0.9
TRAV16 LogFC=−0.9
ENSBTAG00000054381 LogFC=−0.9
FPKM
1hr
3hr
6hr
8hr
24hr
Time Pre−Processing

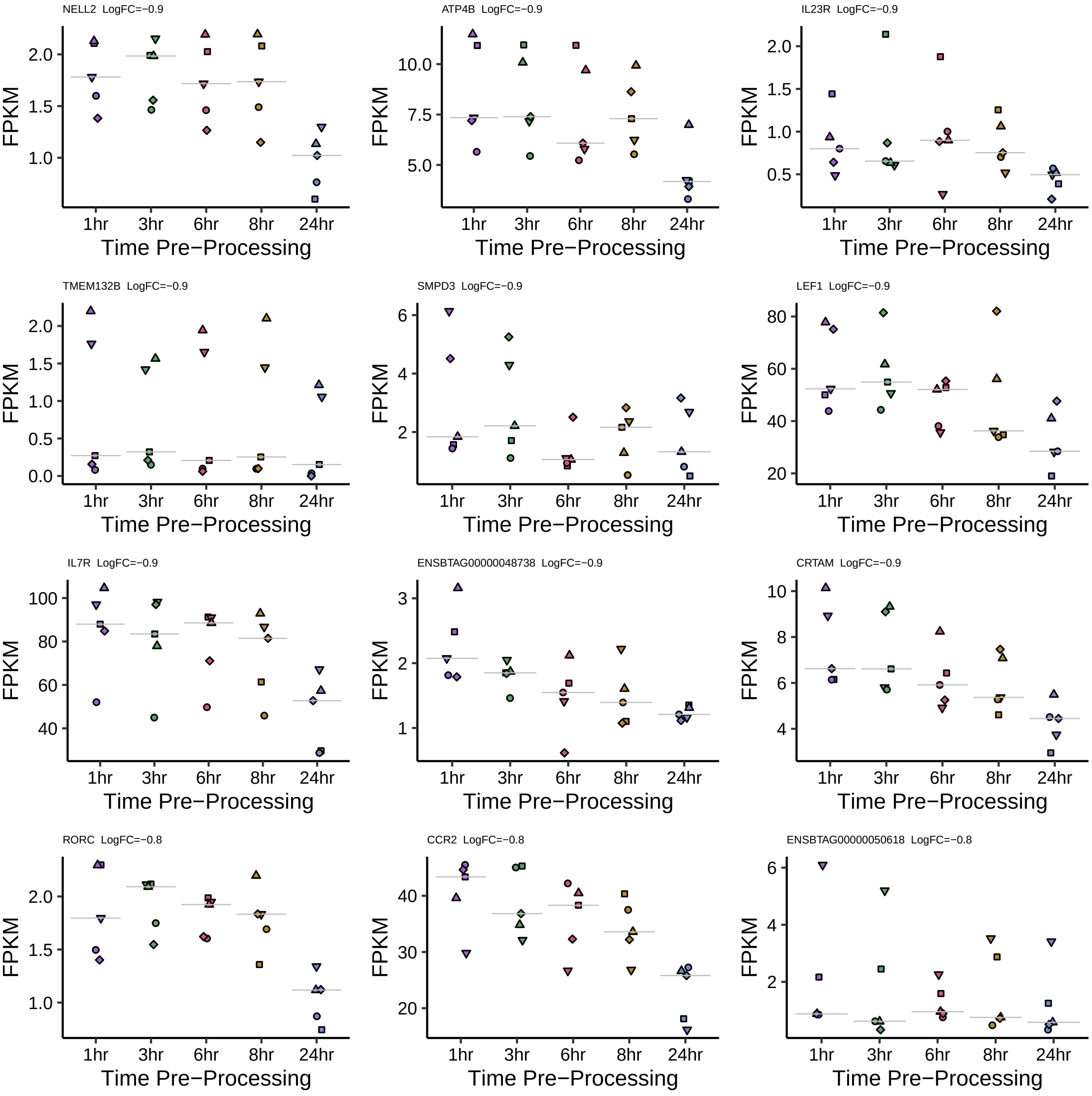
NELL2 LogFC=−0.9
ATP4B LogFC=−0.9
IL23R LogFC=−0.9
TMEM132B LogFC=−0.9
SMPD3 LogFC=−0.9
LEF1 LogFC=−0.9
IL7R LogFC=−0.9
ENSBTAG00000048738 LogFC=−0.9
CRTAM LogFC=−0.9
RORC LogFC=−0.8
CCR2 LogFC=−0.8
ENSBTAG00000050618 LogFC=−0.8
FPKM
1hr
3hr
6hr
8hr
24hr
Time Pre−Processing

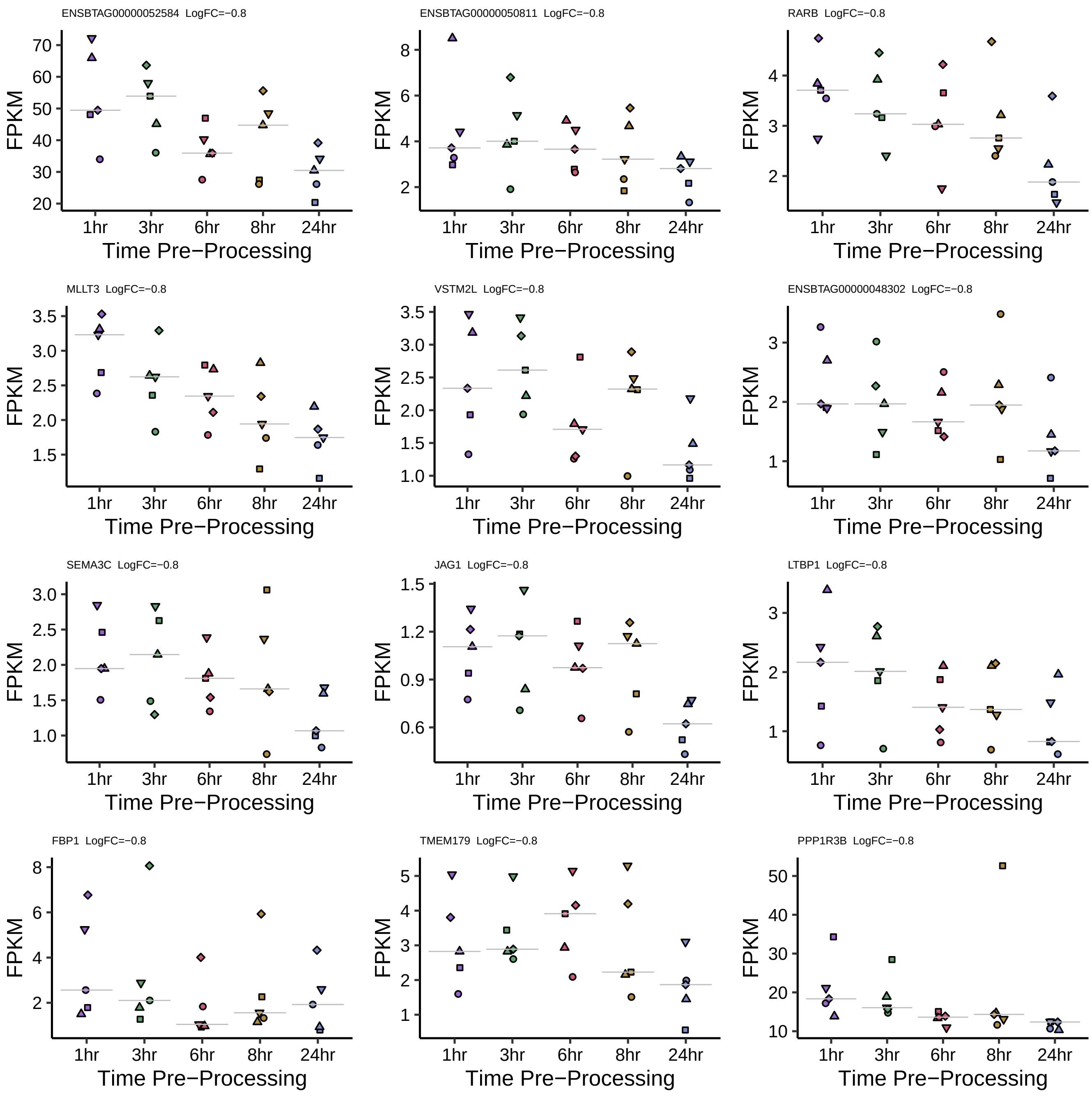

ENSBTAG00000052584 LogFC=−0.8
ENSBTAG00000050811 LogFC=−0.8
RARB LogFC=−0.8
MLLT3 LogFC=−0.8
VSTM2L LogFC=−0.8
ENSBTAG00000048302 LogFC=−0.8
SEMA3C LogFC=−0.8
JAG1 LogFC=−0.8
LTBP1 LogFC=−0.8
FBP1 LogFC=−0.8
TMEM179 LogFC=−0.8
PPP1R3B LogFC=−0.8
FPKM
1hr
3hr
6hr
8hr
24hr
Time Pre−Processing

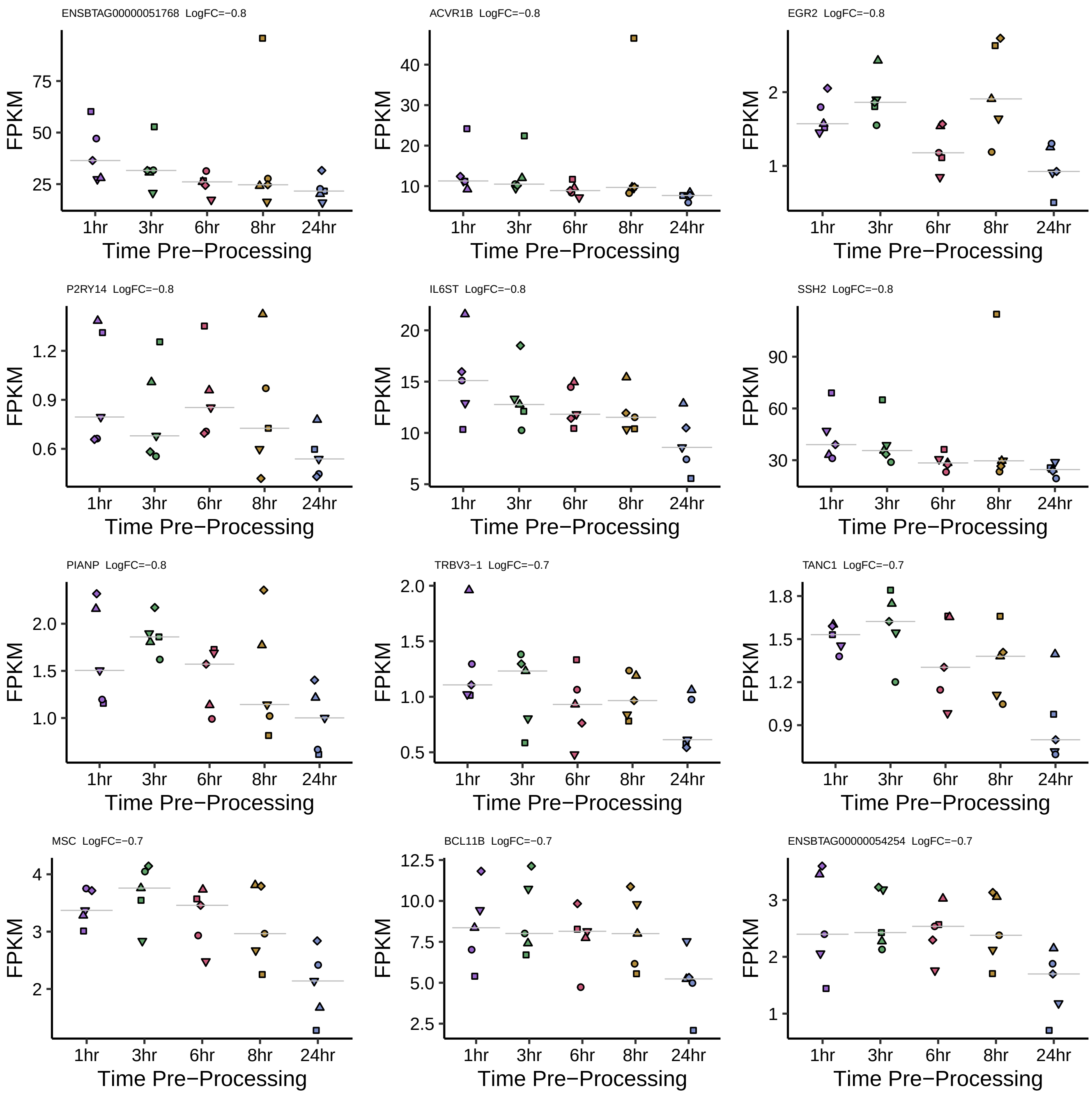
ENSBTAG00000051768 LogFC=−0.8
ACVR1B LogFC=−0.8
EGR2 LogFC=−0.8
P2RY14 LogFC=−0.8
IL6ST LogFC=−0.8
SSH2 LogFC=−0.8
PIANP LogFC=−0.8
TRBV3−1 LogFC=−0.7
TANC1 LogFC=−0.7
MSC LogFC=−0.7
BCL11B LogFC=−0.7
ENSBTAG00000054254 LogFC=−0.7
FPKM
1hr 3hr 6hr 8hr 24hr
Time Pre−Processing

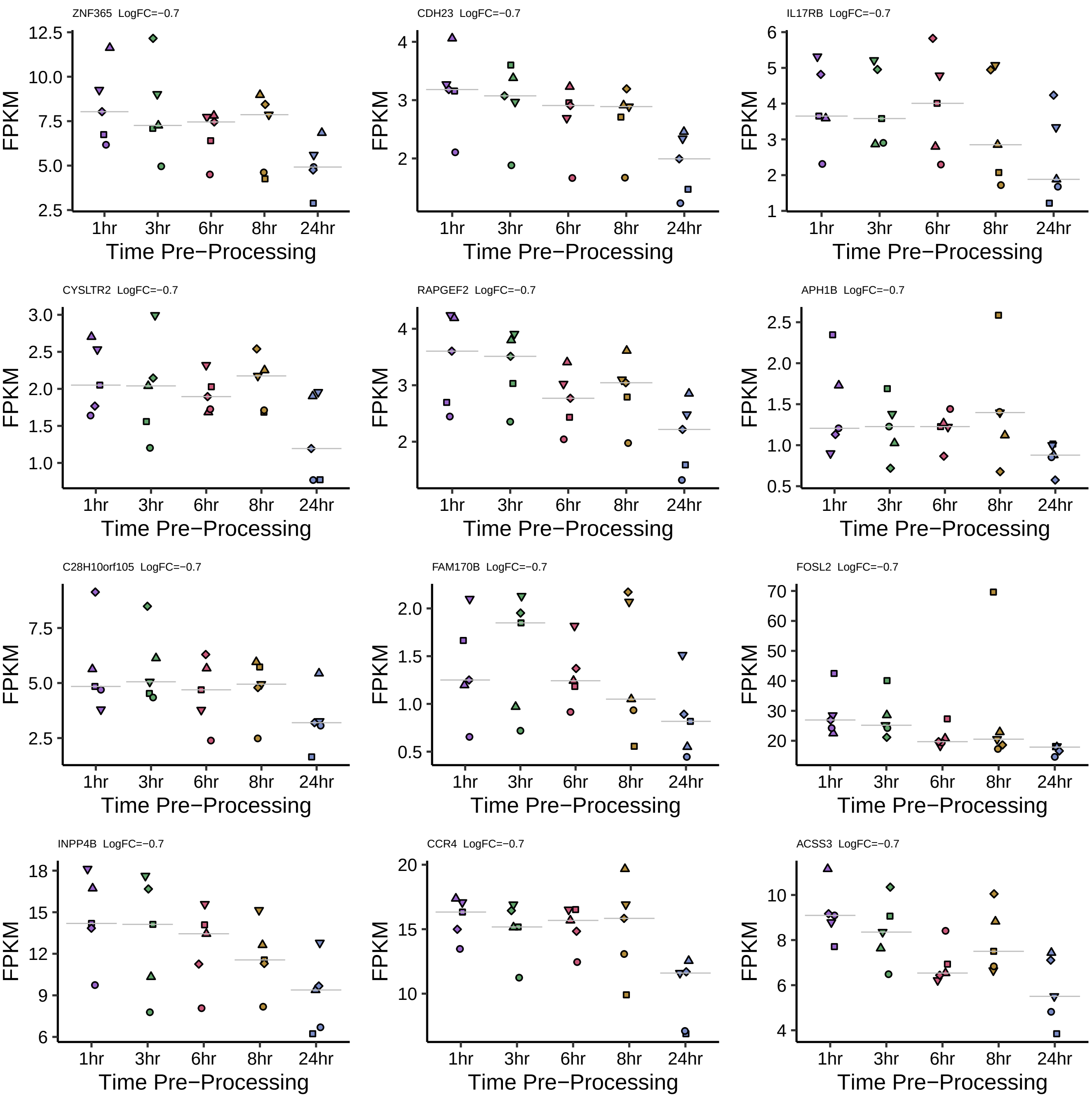

ZNF365 LogFC=−0.7
CDH23 LogFC=−0.7
IL17RB LogFC=−0.7
CYSLTR2 LogFC=−0.7
RAPGEF2 LogFC=−0.7
APH1B LogFC=−0.7
C28H10orf105 LogFC=−0.7
FAM170B LogFC=−0.7
FOSL2 LogFC=−0.7
INPP4B LogFC=−0.7
CCR4 LogFC=−0.7
ACSS3 LogFC=−0.7
FPKM
1hr
3hr
6hr
8hr
24hr
Time Pre−Processing

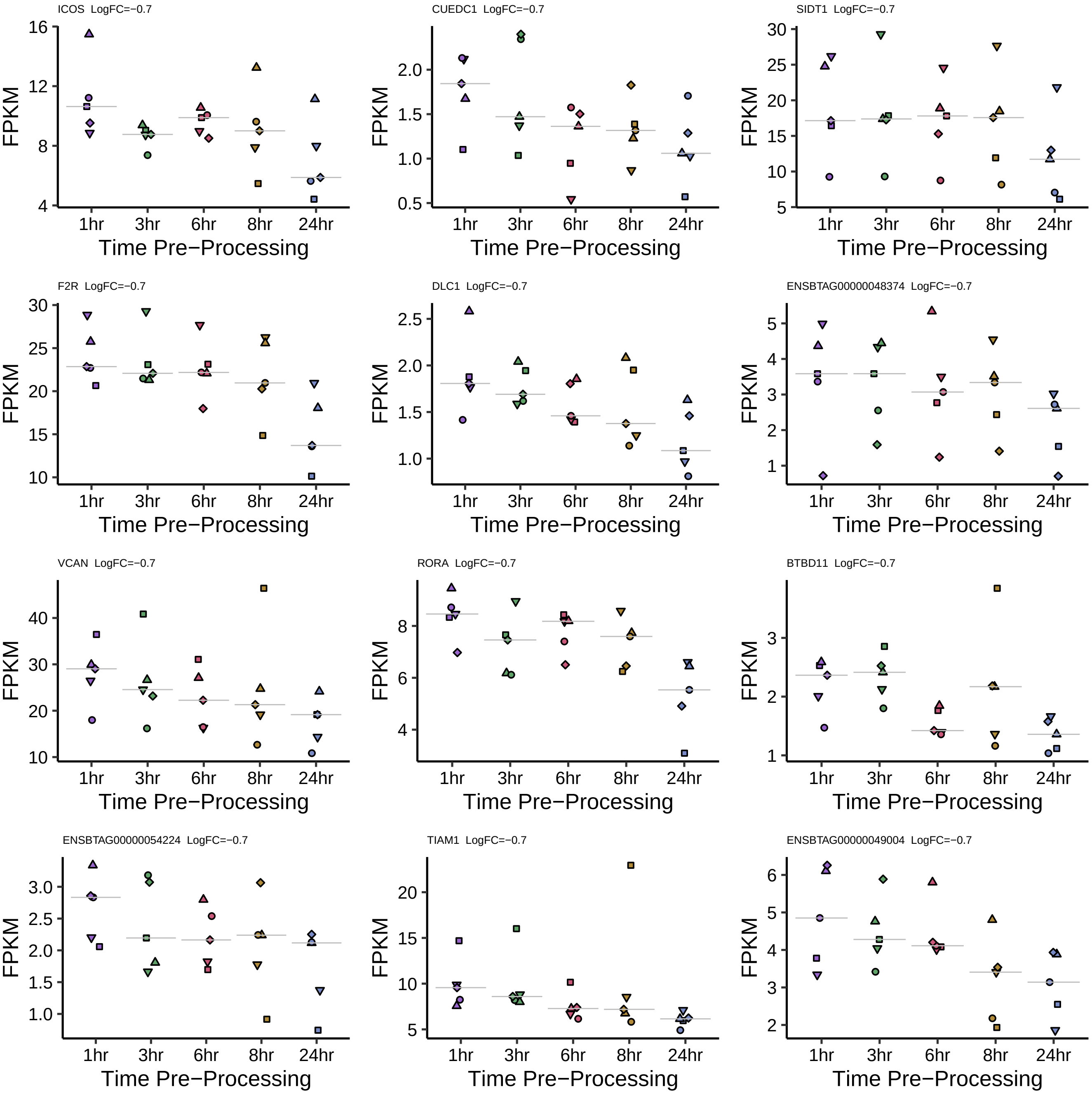
ICOS LogFC=−0.7
CUEDC1 LogFC=−0.7
SIDT1 LogFC=−0.7
F2R LogFC=−0.7
DLC1 LogFC=−0.7
ENSBTAG00000048374 LogFC=−0.7
VCAN LogFC=−0.7
RORA LogFC=−0.7
BTBD11 LogFC=−0.7
ENSBTAG00000054224 LogFC=−0.7
TIAM1 LogFC=−0.7
ENSBTAG00000049004 LogFC=−0.7
FPKM
1hr
3hr
6hr
8hr
24hr
Time Pre−Processing

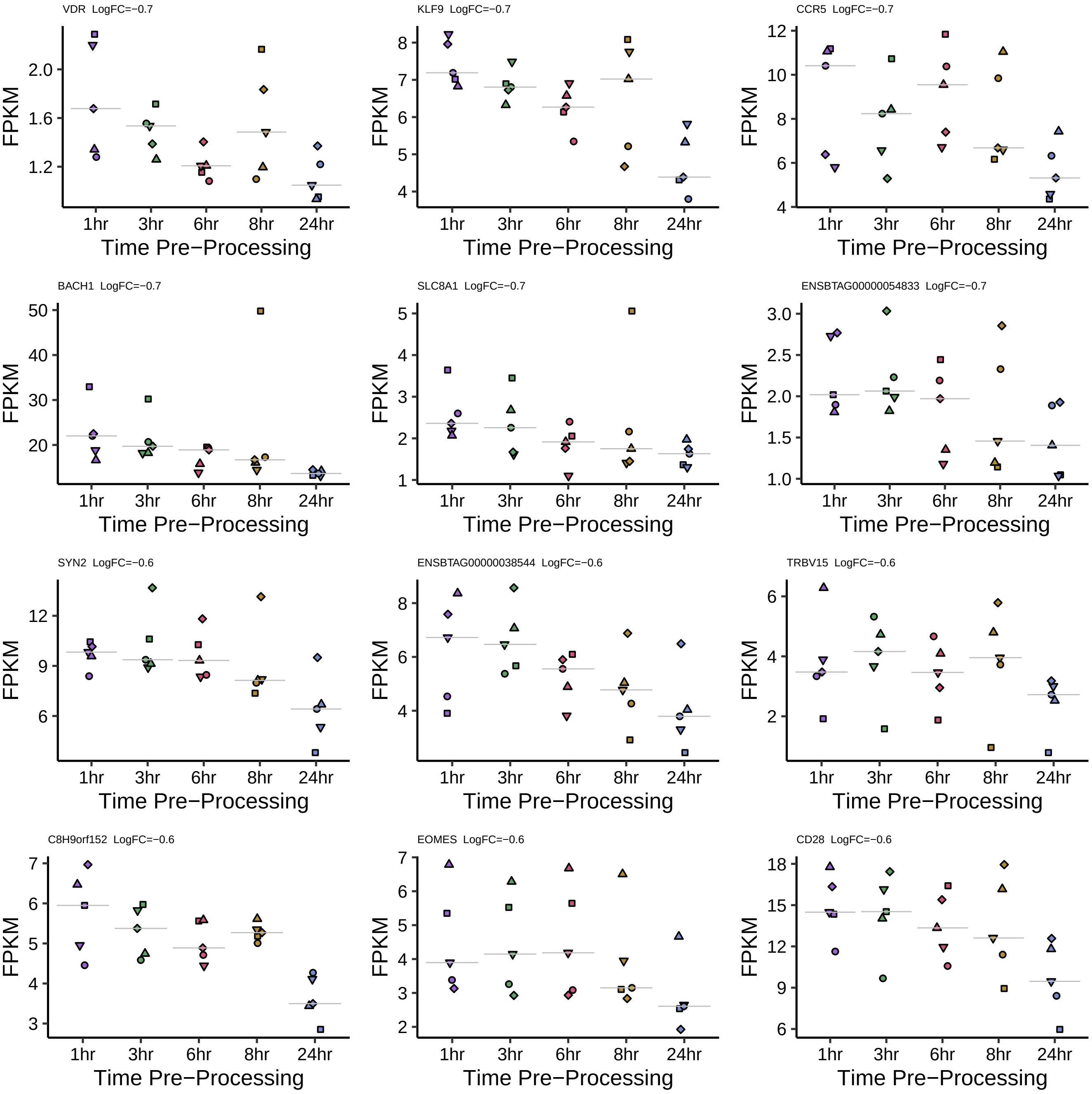

VDR LogFC=−0.7
KLF9 LogFC=−0.7
CCR5 LogFC=−0.7
BACH1 LogFC=−0.7
SLC8A1 LogFC=−0.7
ENSBTAG00000054833 LogFC=−0.7
SYN2 LogFC=−0.6
ENSBTAG00000038544 LogFC=−0.6
TRBV15 LogFC=−0.6
C8H9orf152 LogFC=−0.6
EOMES LogFC=−0.6
CD28 LogFC=−0.6
FPKM
1hr
3hr
6hr
8hr
24hr
Time Pre−Processing

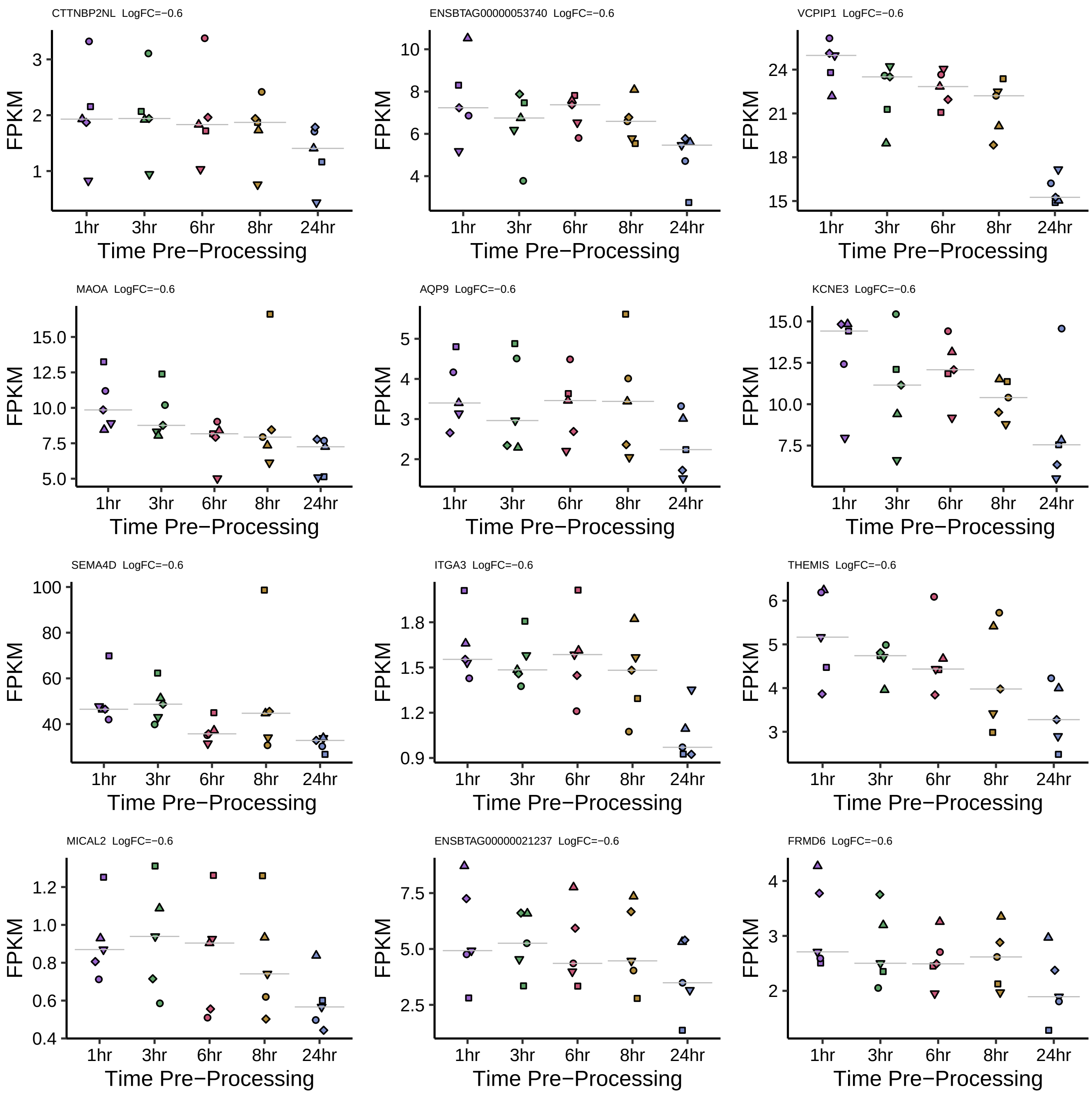
CTTNBP2NL LogFC=−0.6
ENSBTAG00000053740 LogFC=−0.6
VCPIP1 LogFC=−0.6
MAOA LogFC=−0.6
AQP9 LogFC=−0.6
KCNE3 LogFC=−0.6
SEMA4D LogFC=−0.6
ITGA3 LogFC=−0.6
THEMIS LogFC=−0.6
MICAL2 LogFC=−0.6
ENSBTAG00000021237 LogFC=−0.6
FRMD6 LogFC=−0.6
FPKM
1hr
3hr
6hr
8hr
24hr
Time Pre−Processing

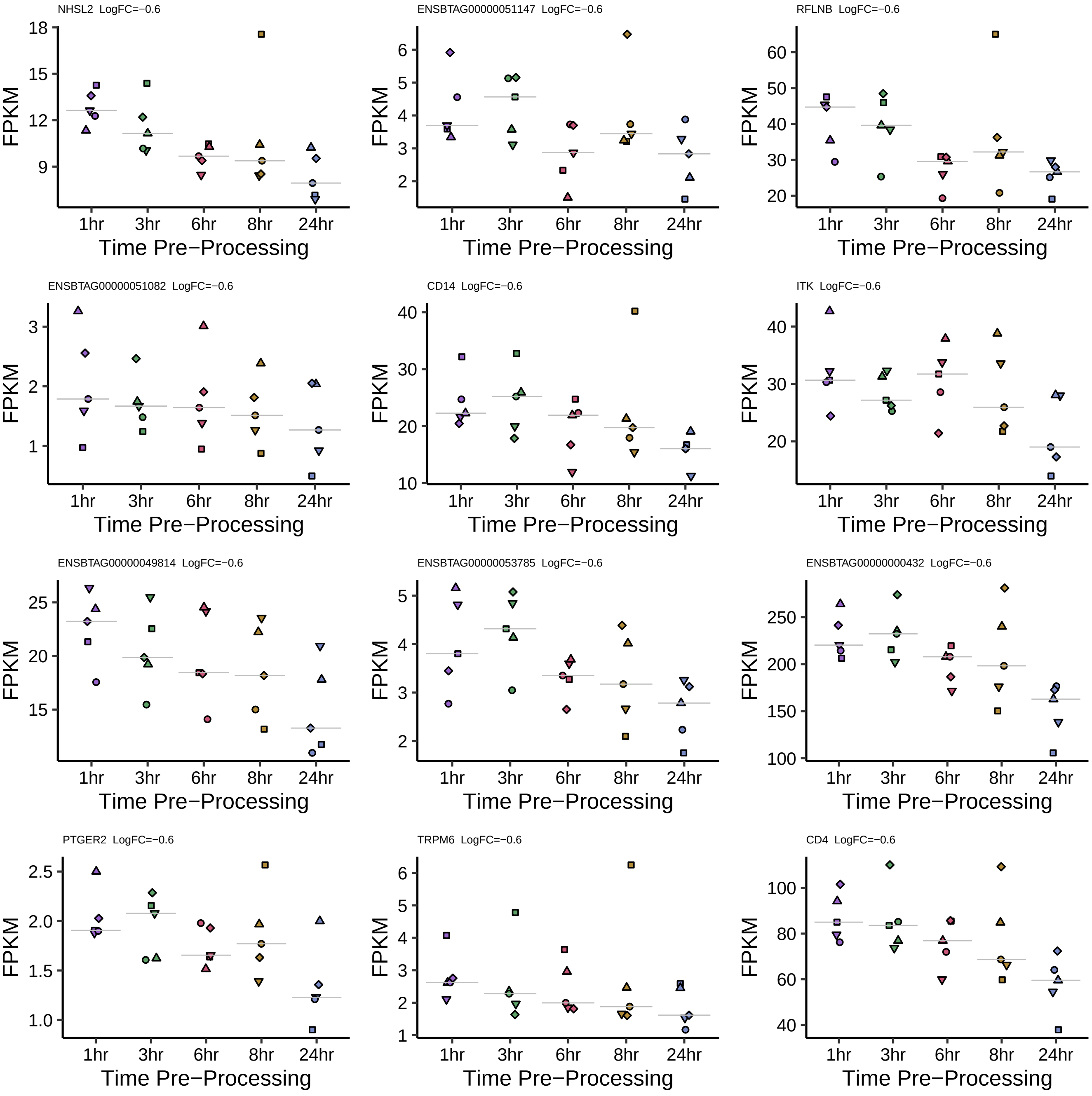
NHSL2 LogFC=−0.6
ENSBTAG00000051147 LogFC=−0.6
RFLNB LogFC=−0.6
ENSBTAG00000051082 LogFC=−0.6
CD14 LogFC=−0.6
ITK LogFC=−0.6
ENSBTAG00000049814 LogFC=−0.6
ENSBTAG00000053785 LogFC=−0.6
ENSBTAG00000000432 LogFC=−0.6
PTGER2 LogFC=−0.6
TRPM6 LogFC=−0.6
CD4 LogFC=−0.6
FPKM
1hr
3hr
6hr
8hr
24hr
Time Pre−Processing

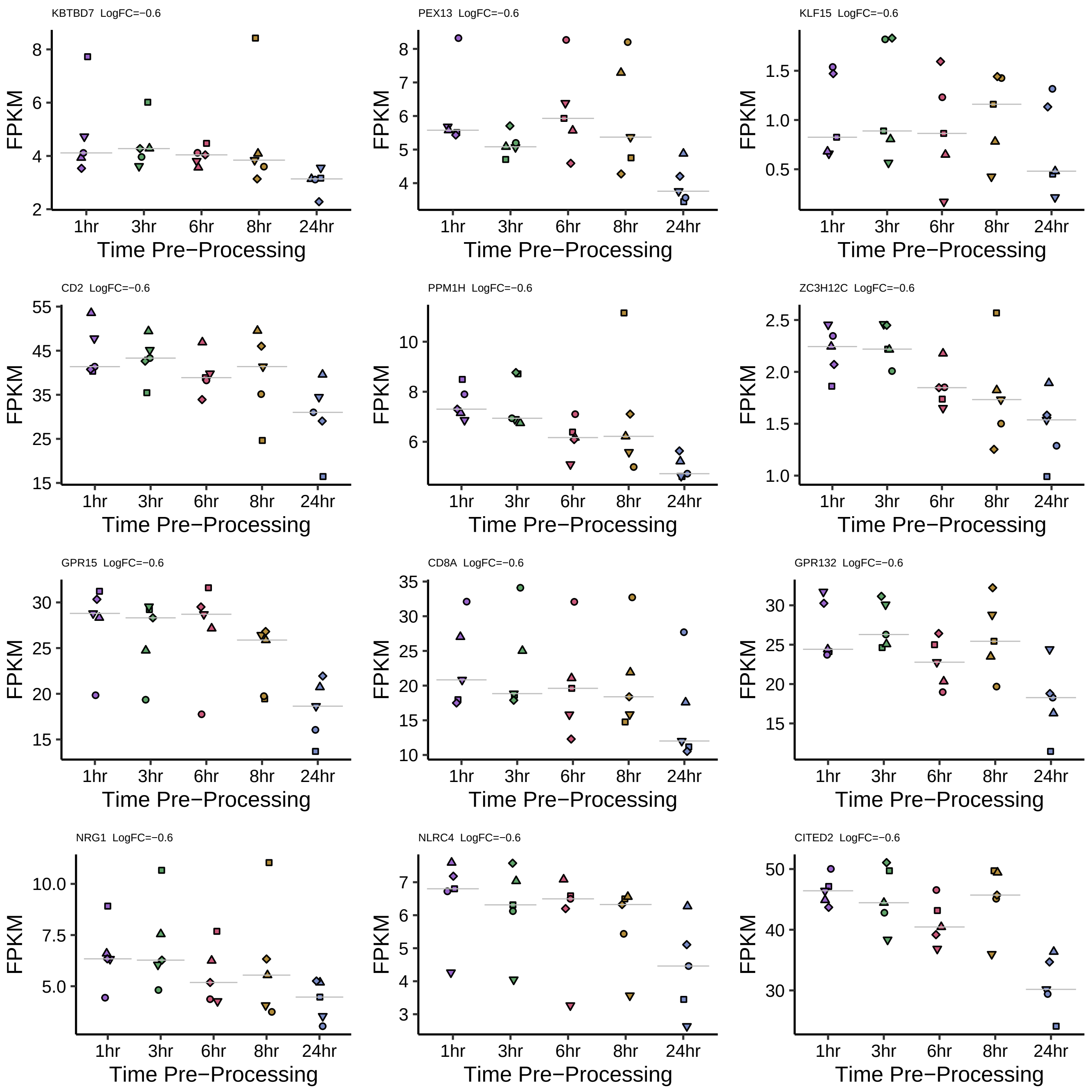
KBTBD7 LogFC=−0.6
PEX13 LogFC=−0.6
KLF15 LogFC=−0.6
CD2 LogFC=−0.6
PPM1H LogFC=−0.6
ZC3H12C LogFC=−0.6
GPR15 LogFC=−0.6
CD8A LogFC=−0.6
GPR132 LogFC=−0.6
NRG1 LogFC=−0.6
NLRC4 LogFC=−0.6
CITED2 LogFC=−0.6
FPKM
1hr 3hr 6hr 8hr 24hr
Time Pre−Processing

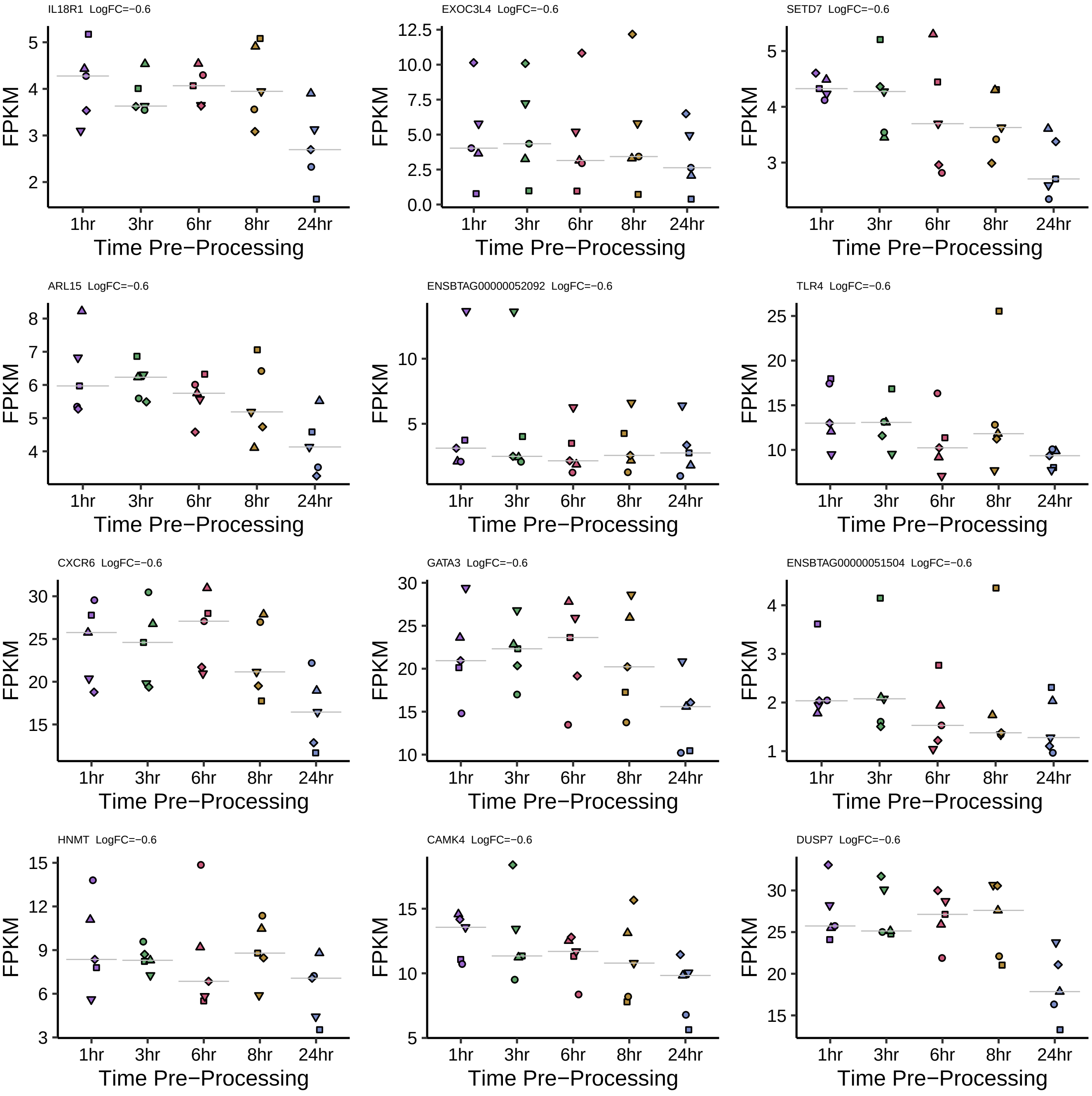
IL18R1 LogFC=−0.6
EXOC3L4 LogFC=−0.6
SETD7 LogFC=−0.6
ARL15 LogFC=−0.6
ENSBTAG00000052092 LogFC=−0.6
TLR4 LogFC=−0.6
CXCR6 LogFC=−0.6
GATA3 LogFC=−0.6
ENSBTAG00000051504 LogFC=−0.6
HNMT LogFC=−0.6
CAMK4 LogFC=−0.6
DUSP7 LogFC=−0.6
FPKM
1hr
3hr
6hr
8hr
24hr
Time Pre−Processing

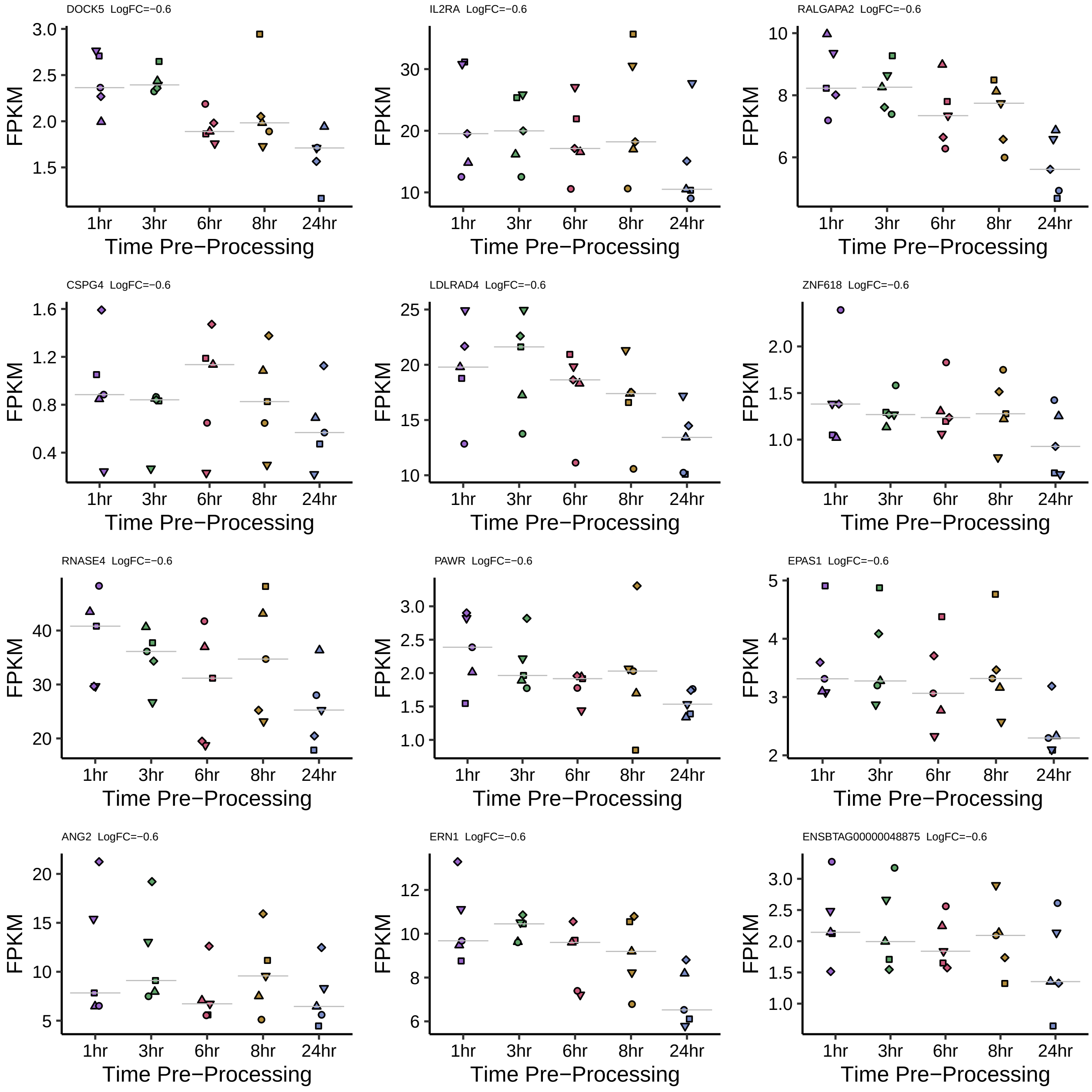

DOCK5 LogFC=−0.6
IL2RA LogFC=−0.6
RALGAPA2 LogFC=−0.6
CSPG4 LogFC=−0.6
LDLRAD4 LogFC=−0.6
ZNF618 LogFC=−0.6
RNASE4 LogFC=−0.6
PAWR LogFC=−0.6
EPAS1 LogFC=−0.6
ANG2 LogFC=−0.6
ERN1 LogFC=−0.6
ENSBTAG00000048875 LogFC=−0.6
FPKM
Time Pre−Processing
1hr
3hr
6hr
8hr
24hr

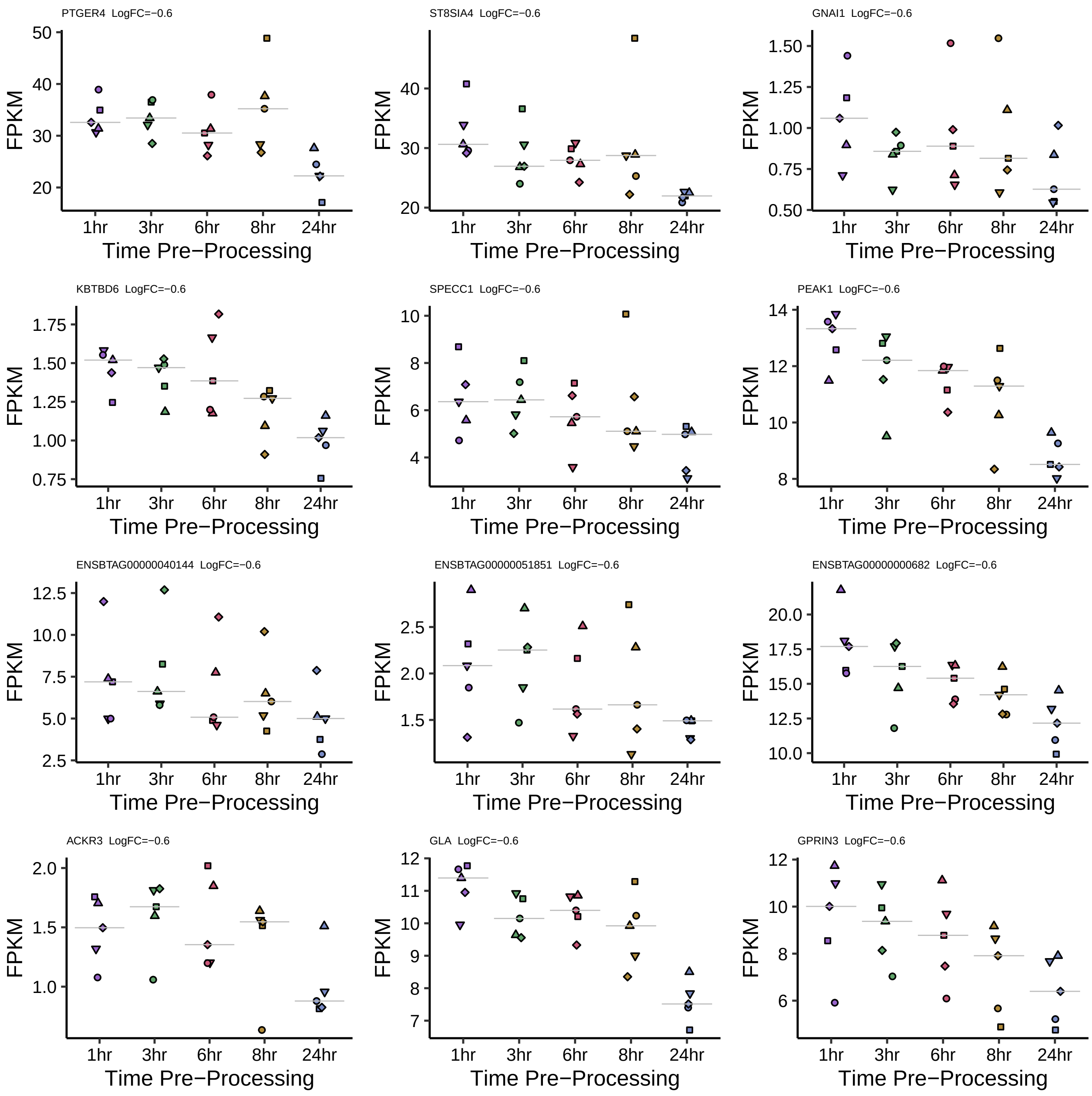
PTGER4 LogFC=−0.6
ST8SIA4 LogFC=−0.6
GNAI1 LogFC=−0.6
KBTBD6 LogFC=−0.6
SPECC1 LogFC=−0.6
PEAK1 LogFC=−0.6
ENSBTAG00000040144 LogFC=−0.6
ENSBTAG00000051851 LogFC=−0.6
ENSBTAG00000000682 LogFC=−0.6
ACKR3 LogFC=−0.6
GLA LogFC=−0.6
GPRIN3 LogFC=−0.6
FPKM
1hr
3hr
6hr
8hr
24hr
Time Pre−Processing

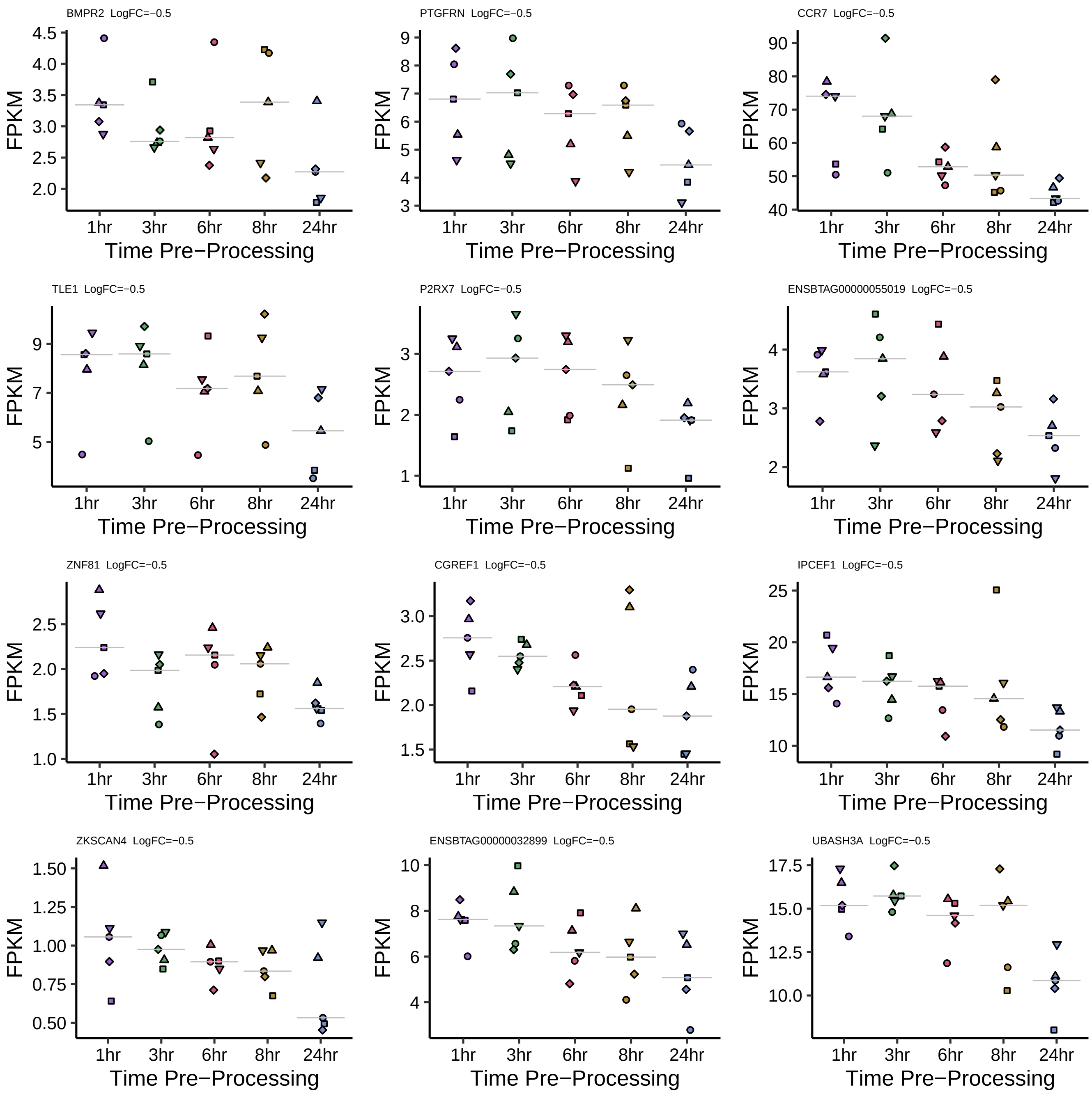
BMPR2 LogFC=−0.5
PTGFRN LogFC=−0.5
CCR7 LogFC=−0.5
TLE1 LogFC=−0.5
P2RX7 LogFC=−0.5
ENSBTAG00000055019 LogFC=−0.5
ZNF81 LogFC=−0.5
CGREF1 LogFC=−0.5
IPCEF1 LogFC=−0.5
ZKSCAN4 LogFC=−0.5
ENSBTAG00000032899 LogFC=−0.5
UBASH3A LogFC=−0.5
FPKM
1hr 3hr 6hr 8hr 24hr
Time Pre−Processing

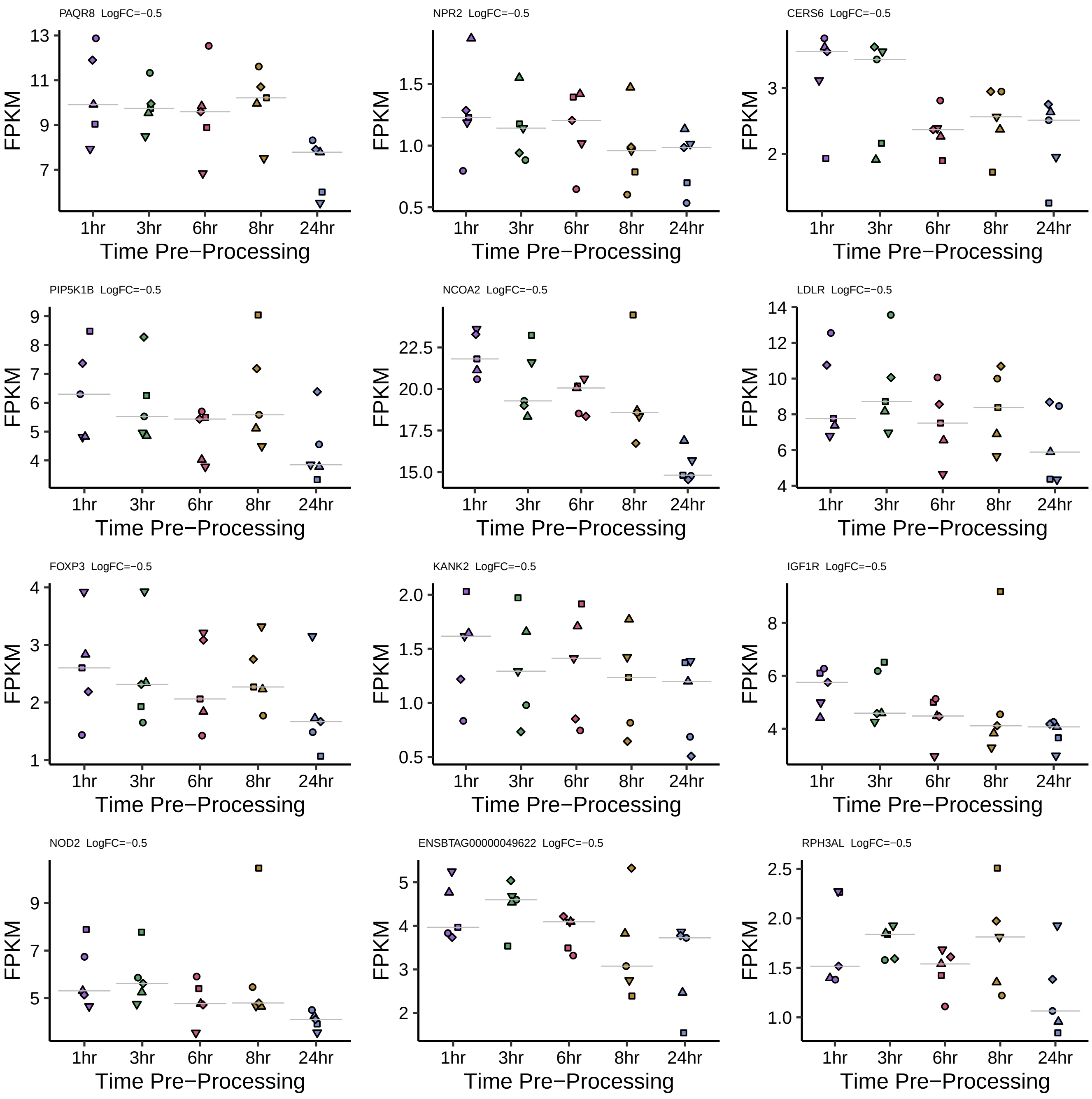

PAQR8 LogFC=−0.5
NPR2 LogFC=−0.5
CERS6 LogFC=−0.5
PIP5K1B LogFC=−0.5
NCOA2 LogFC=−0.5
LDLR LogFC=−0.5
FOXP3 LogFC=−0.5
KANK2 LogFC=−0.5
IGF1R LogFC=−0.5
NOD2 LogFC=−0.5
ENSBTAG00000049622 LogFC=−0.5
RPH3AL LogFC=−0.5
FPKM
1hr 3hr 6hr 8hr 24hr
Time Pre−Processing

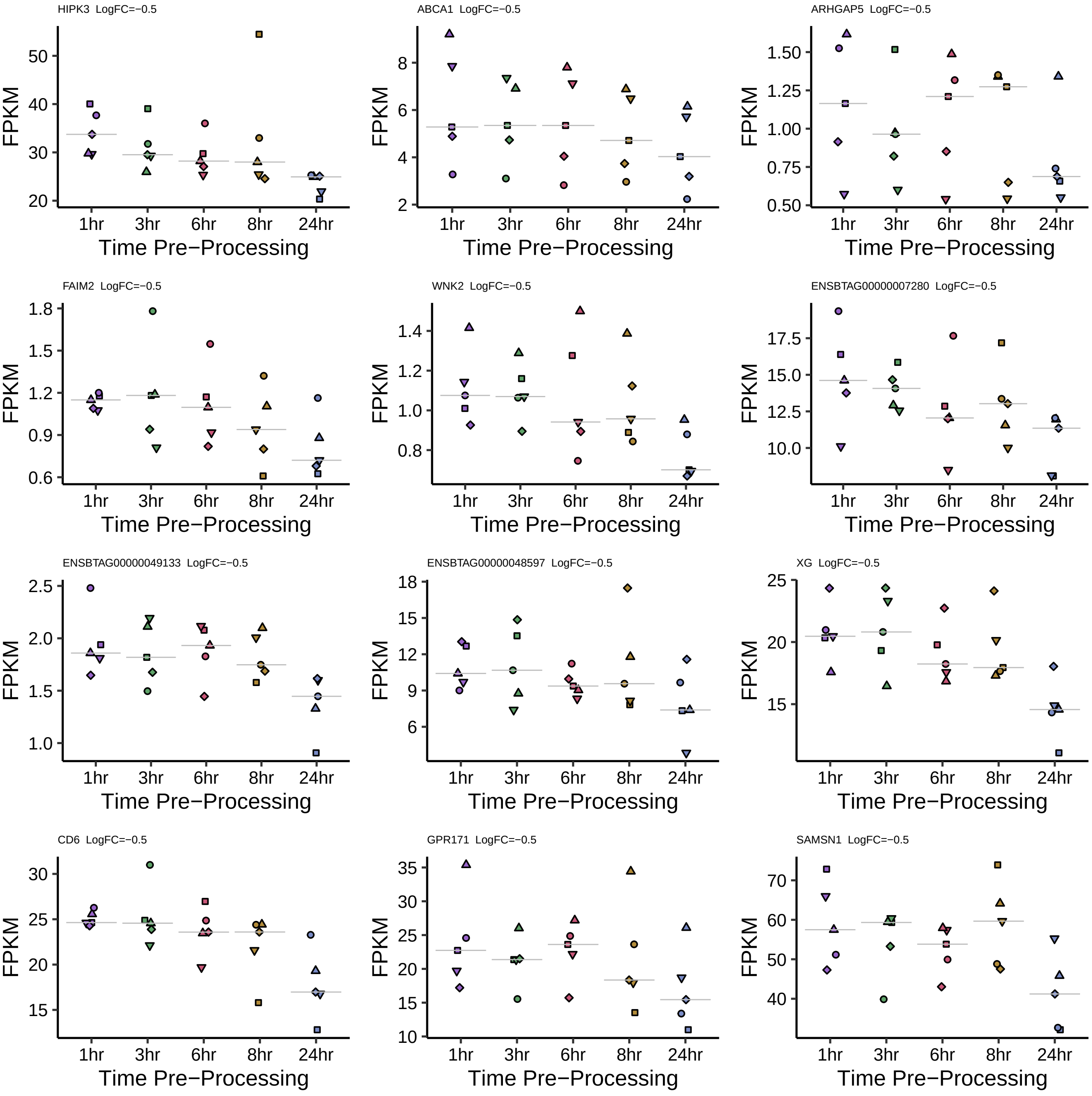
HIPK3 LogFC=−0.5
ABCA1 LogFC=−0.5
ARHGAP5 LogFC=−0.5
FAIM2 LogFC=−0.5
WNK2 LogFC=−0.5
ENSBTAG00000007280 LogFC=−0.5
ENSBTAG00000049133 LogFC=−0.5
ENSBTAG00000048597 LogFC=−0.5
XG LogFC=−0.5
CD6 LogFC=−0.5
GPR171 LogFC=−0.5
SAMSN1 LogFC=−0.5
FPKM
1hr
3hr
6hr
8hr
24hr
Time Pre−Processing

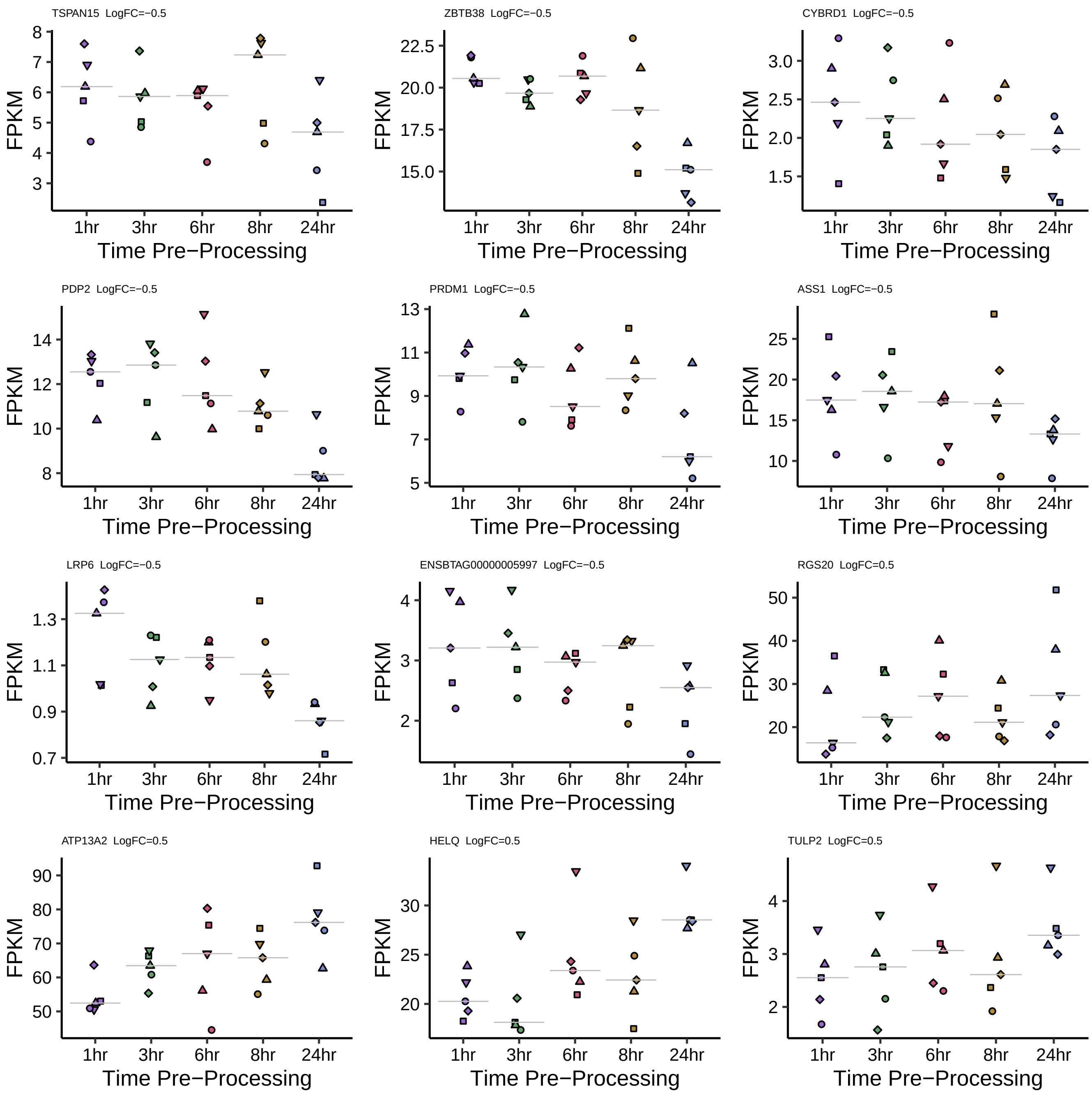

TSPAN15 LogFC=−0.5
ZBTB38 LogFC=−0.5
CYBRD1 LogFC=−0.5
PDP2 LogFC=−0.5
PRDM1 LogFC=−0.5
ASS1 LogFC=−0.5
LRP6 LogFC=−0.5
ENSBTAG00000005997 LogFC=−0.5
RGS20 LogFC=0.5
ATP13A2 LogFC=0.5
HELQ LogFC=0.5
TULP2 LogFC=0.5
FPKM
1hr
3hr
6hr
8hr
24hr
Time Pre−Processing

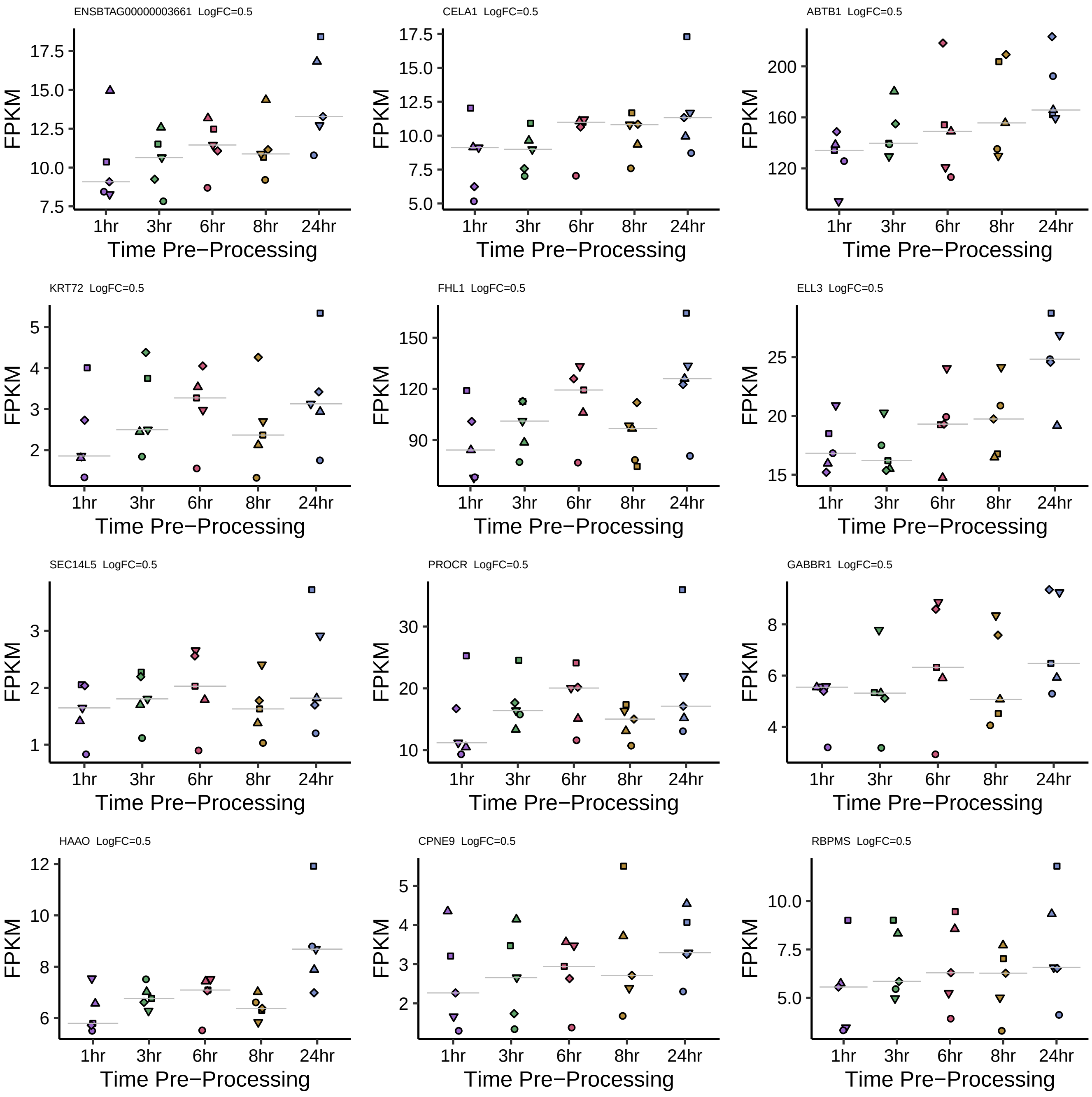

ENSBTAG00000003661 LogFC=0.5
CELA1 LogFC=0.5
ABTB1 LogFC=0.5
KRT72 LogFC=0.5
FHL1 LogFC=0.5
ELL3 LogFC=0.5
SEC14L5 LogFC=0.5
PROCR LogFC=0.5
GABBR1 LogFC=0.5
HAAO LogFC=0.5
CPNE9 LogFC=0.5
RBPMS LogFC=0.5
FPKM
1hr
3hr
6hr
8hr
24hr
Time Pre−Processing

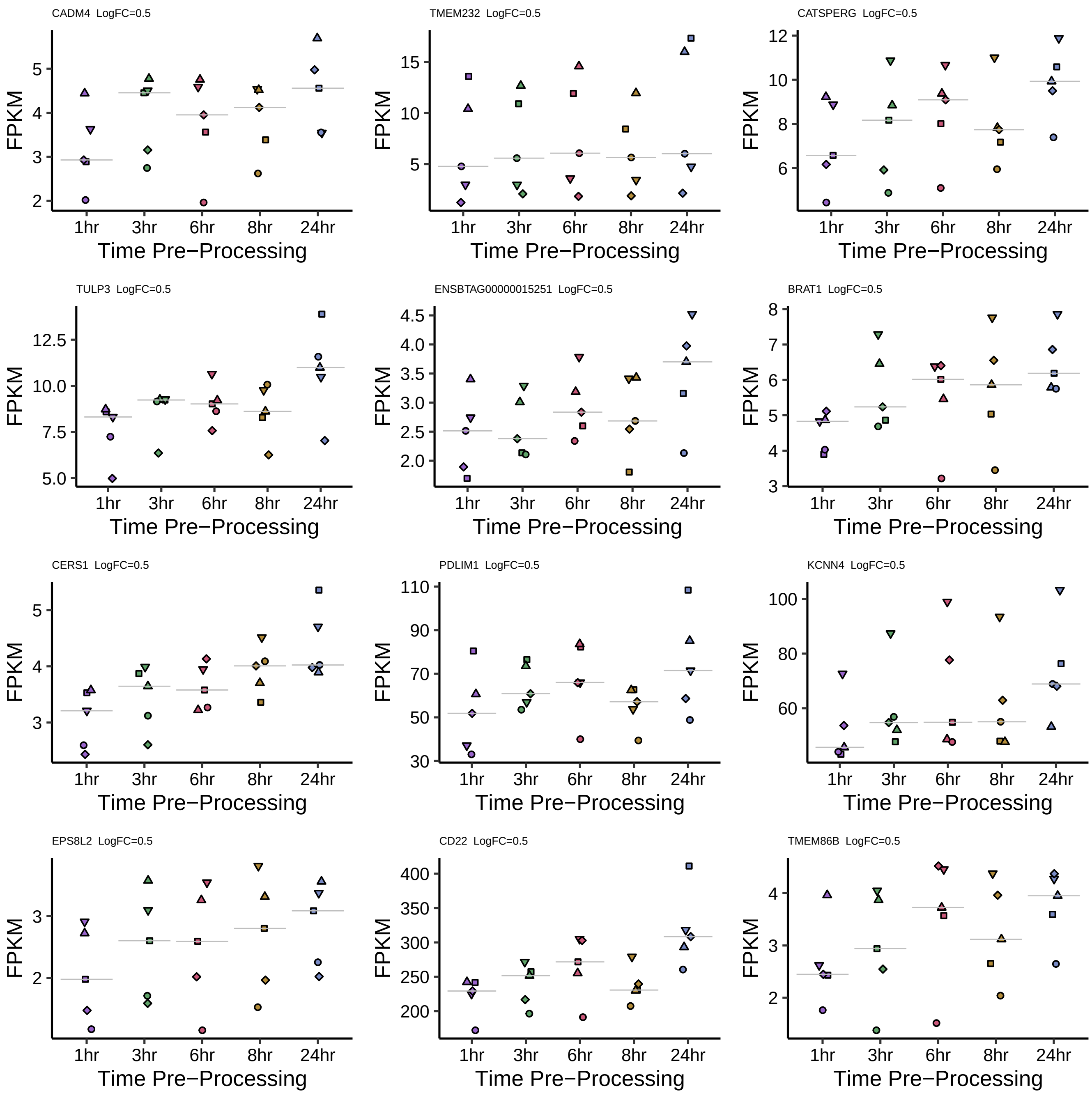
CADM4 LogFC=0.5
TMEM232 LogFC=0.5
CATSPERG LogFC=0.5
TULP3 LogFC=0.5
ENSBTAG00000015251 LogFC=0.5
BRAT1 LogFC=0.5
CERS1 LogFC=0.5
PDLIM1 LogFC=0.5
KCNN4 LogFC=0.5
EPS8L2 LogFC=0.5
CD22 LogFC=0.5
TMEM86B LogFC=0.5
FPKM
1hr 3hr 6hr 8hr 24hr
Time Pre−Processing

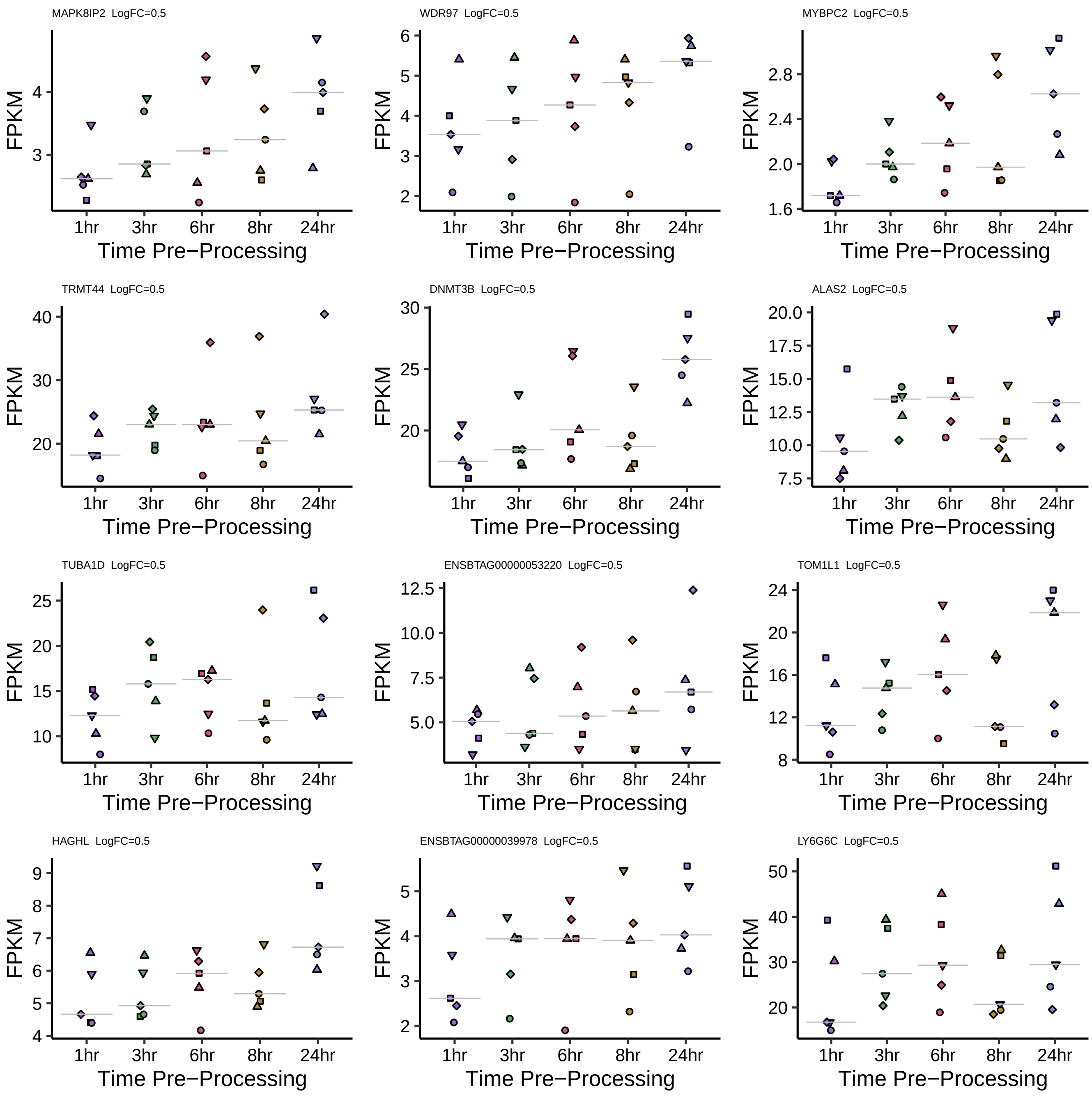
MAPK8IP2 LogFC=0.5
WDR97 LogFC=0.5
MYBPC2 LogFC=0.5
TRMT44 LogFC=0.5
DNMT3B LogFC=0.5
ALAS2 LogFC=0.5
TUBA1D LogFC=0.5
ENSBTAG00000053220 LogFC=0.5
TOM1L1 LogFC=0.5
HAGHL LogFC=0.5
ENSBTAG00000039978 LogFC=0.5
LY6G6C LogFC=0.5
FPKM
1hr
3hr
6hr
8hr
24hr
Time Pre−Processing

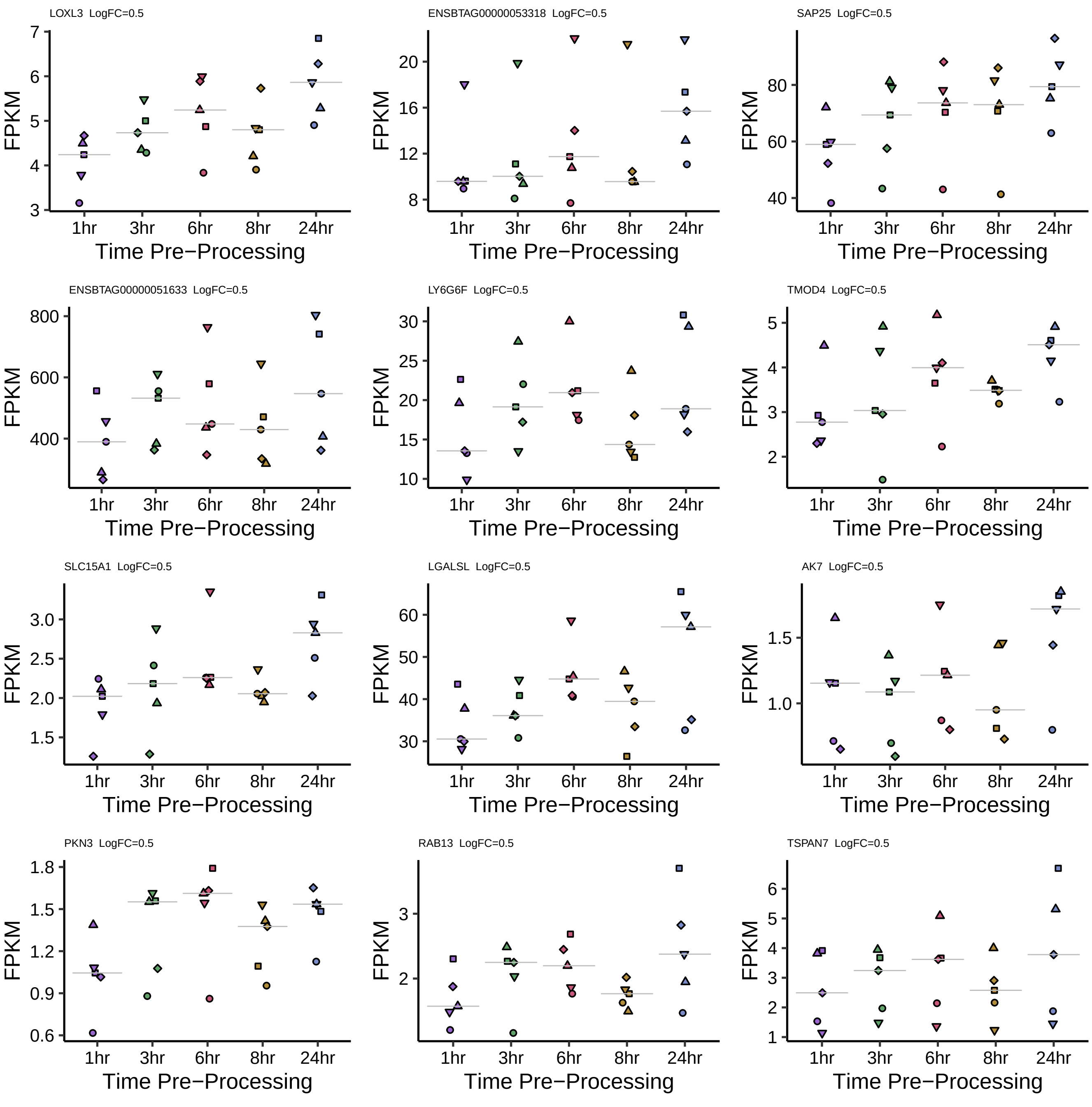

LOXL3 LogFC=0.5
ENSBTAG00000053318 LogFC=0.5
SAP25 LogFC=0.5
ENSBTAG00000051633 LogFC=0.5
LY6G6F LogFC=0.5
TMOD4 LogFC=0.5
SLC15A1 LogFC=0.5
LGALSL LogFC=0.5
AK7 LogFC=0.5
PKN3 LogFC=0.5
RAB13 LogFC=0.5
TSPAN7 LogFC=0.5
FPKM
1hr 3hr 6hr 8hr 24hr
Time Pre−Processing

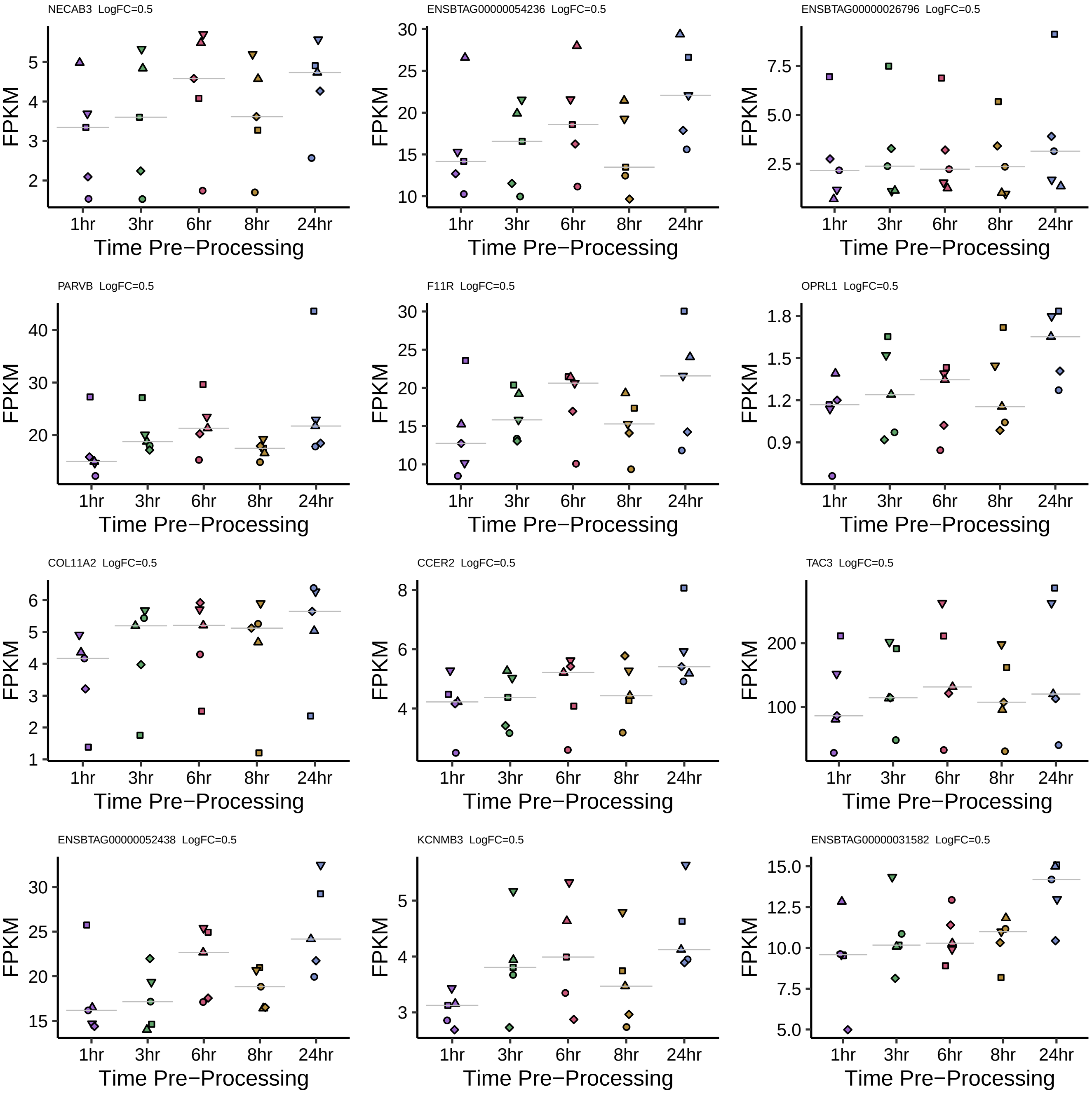
NECAB3 LogFC=0.5
ENSBTAG00000054236 LogFC=0.5
ENSBTAG00000026796 LogFC=0.5
PARVB LogFC=0.5
F11R LogFC=0.5
OPRL1 LogFC=0.5
COL11A2 LogFC=0.5
CCER2 LogFC=0.5
TAC3 LogFC=0.5
ENSBTAG00000052438 LogFC=0.5
KCNMB3 LogFC=0.5
ENSBTAG00000031582 LogFC=0.5
FPKM
1hr
3hr
6hr
8hr
24hr
Time Pre−Processing

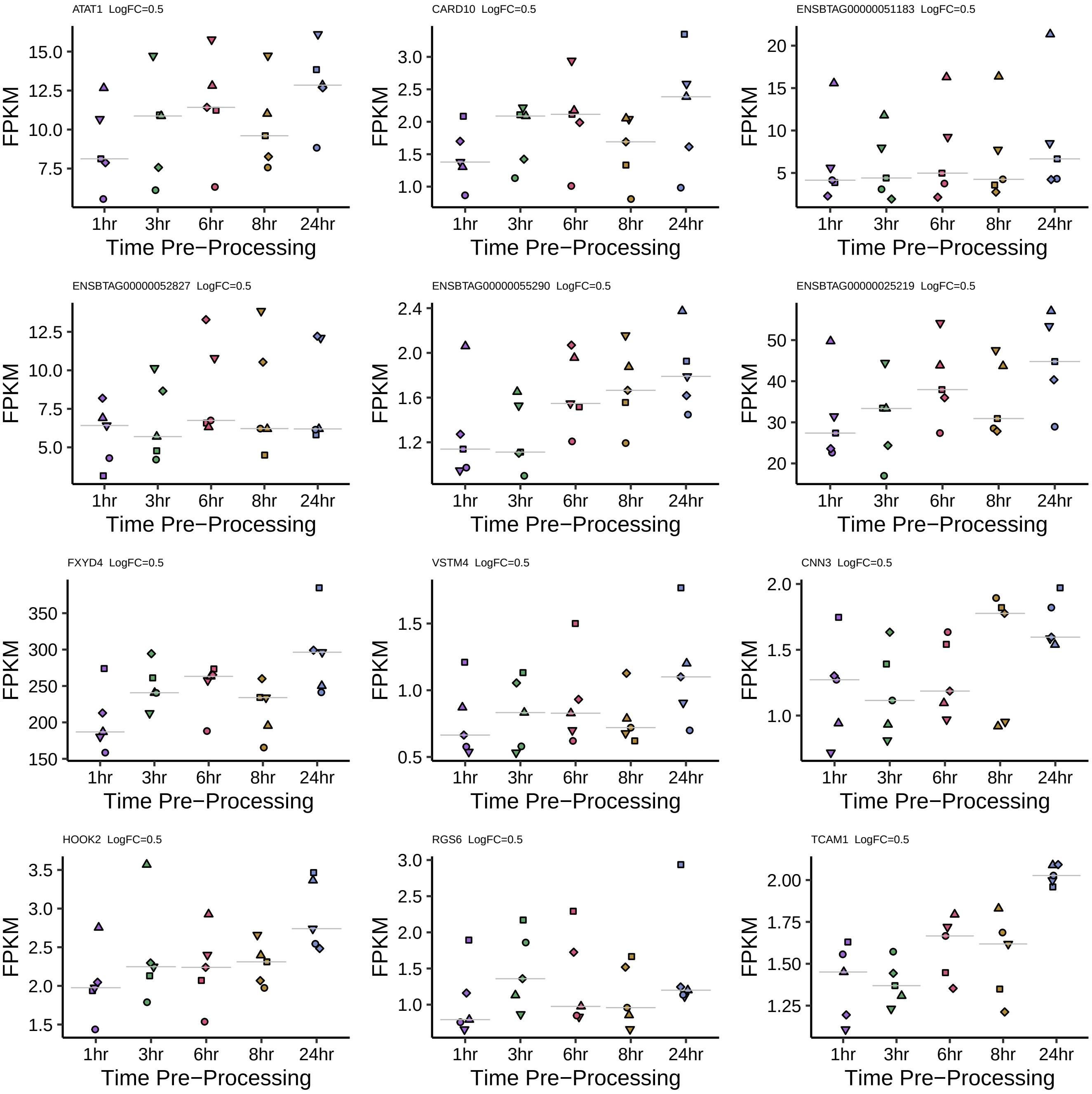
ATAT1 LogFC=0.5
CARD10 LogFC=0.5
ENSBTAG00000051183 LogFC=0.5
ENSBTAG00000052827 LogFC=0.5
ENSBTAG00000055290 LogFC=0.5
ENSBTAG00000025219 LogFC=0.5
FXYD4 LogFC=0.5
VSTM4 LogFC=0.5
CNN3 LogFC=0.5
HOOK2 LogFC=0.5
RGS6 LogFC=0.5
TCAM1 LogFC=0.5
FPKM
1hr
3hr
6hr
8hr
24hr
Time Pre−Processing

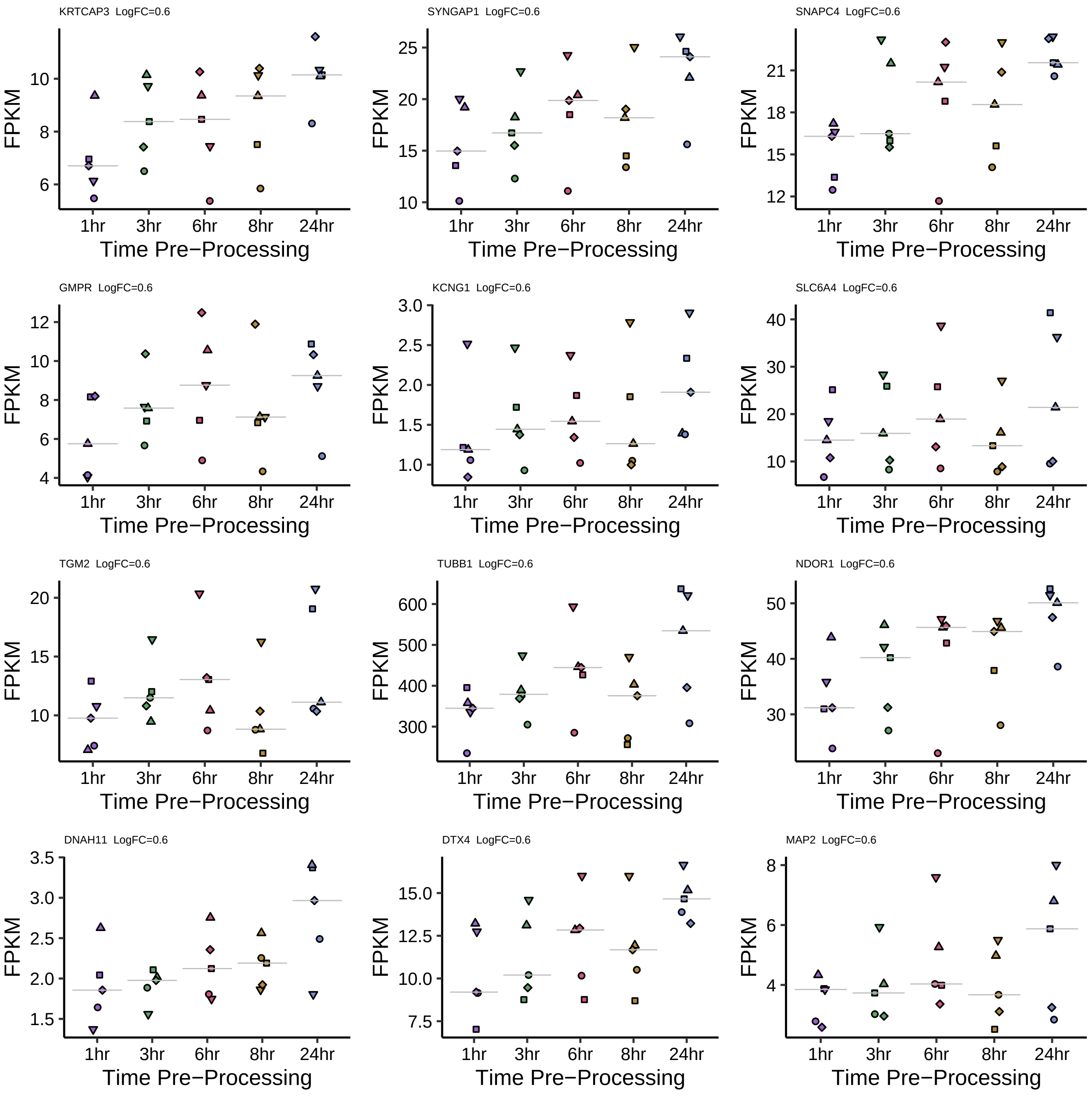
KRTCAP3 LogFC=0.6
SYNGAP1 LogFC=0.6
SNAPC4 LogFC=0.6
GMPR LogFC=0.6
KCNG1 LogFC=0.6
SLC6A4 LogFC=0.6
TGM2 LogFC=0.6
TUBB1 LogFC=0.6
NDOR1 LogFC=0.6
DNAH11 LogFC=0.6
DTX4 LogFC=0.6
MAP2 LogFC=0.6
FPKM
1hr
3hr
6hr
8hr
24hr
Time Pre−Processing

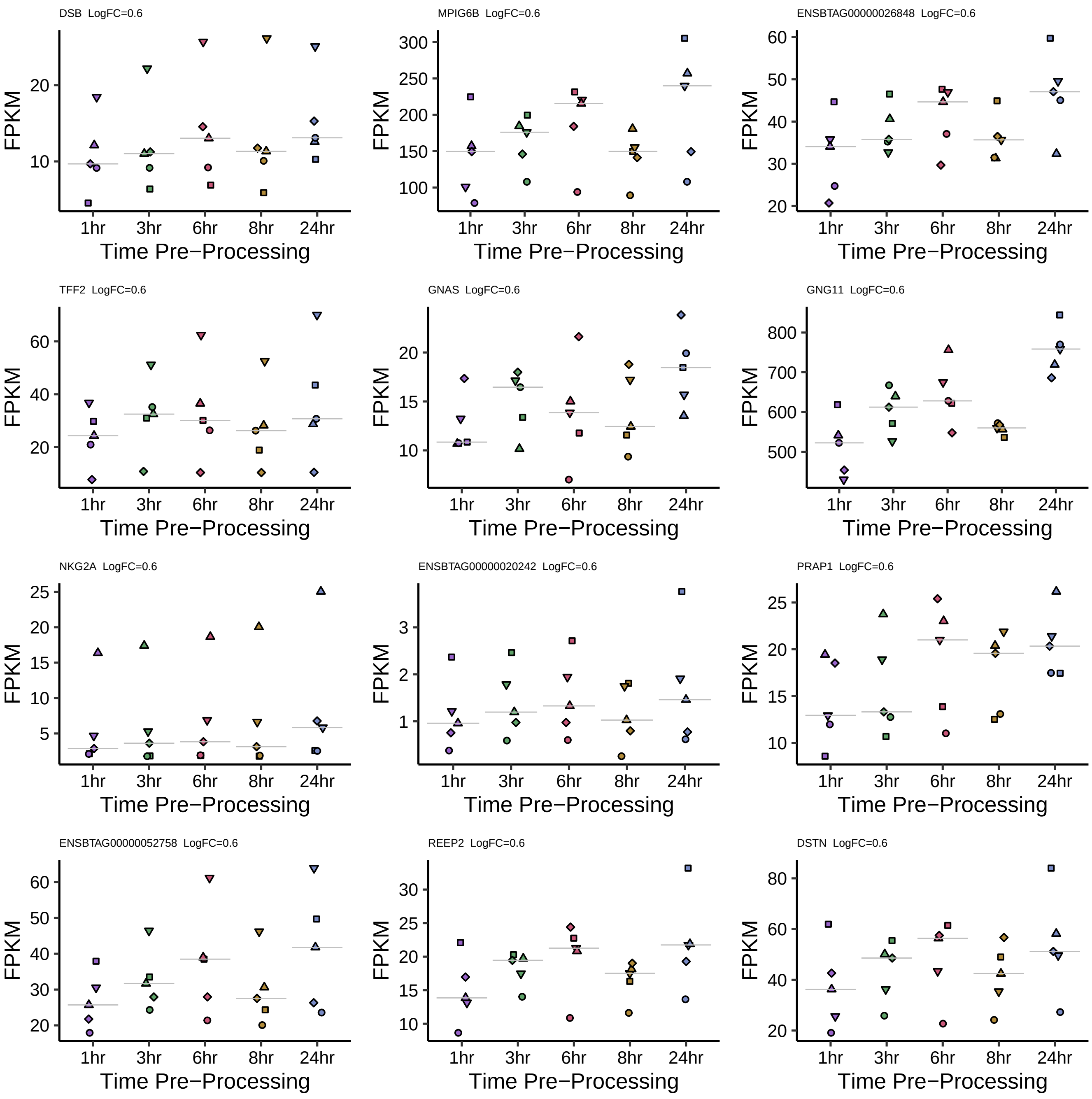
DSB LogFC=0.6
MPIG6B LogFC=0.6
ENSBTAG00000026848 LogFC=0.6
TFF2 LogFC=0.6
GNAS LogFC=0.6
GNG11 LogFC=0.6
NKG2A LogFC=0.6
ENSBTAG00000020242 LogFC=0.6
PRAP1 LogFC=0.6
ENSBTAG00000052758 LogFC=0.6
REEP2 LogFC=0.6
DSTN LogFC=0.6
FPKM
1hr 3hr 6hr 8hr 24hr
Time Pre−Processing

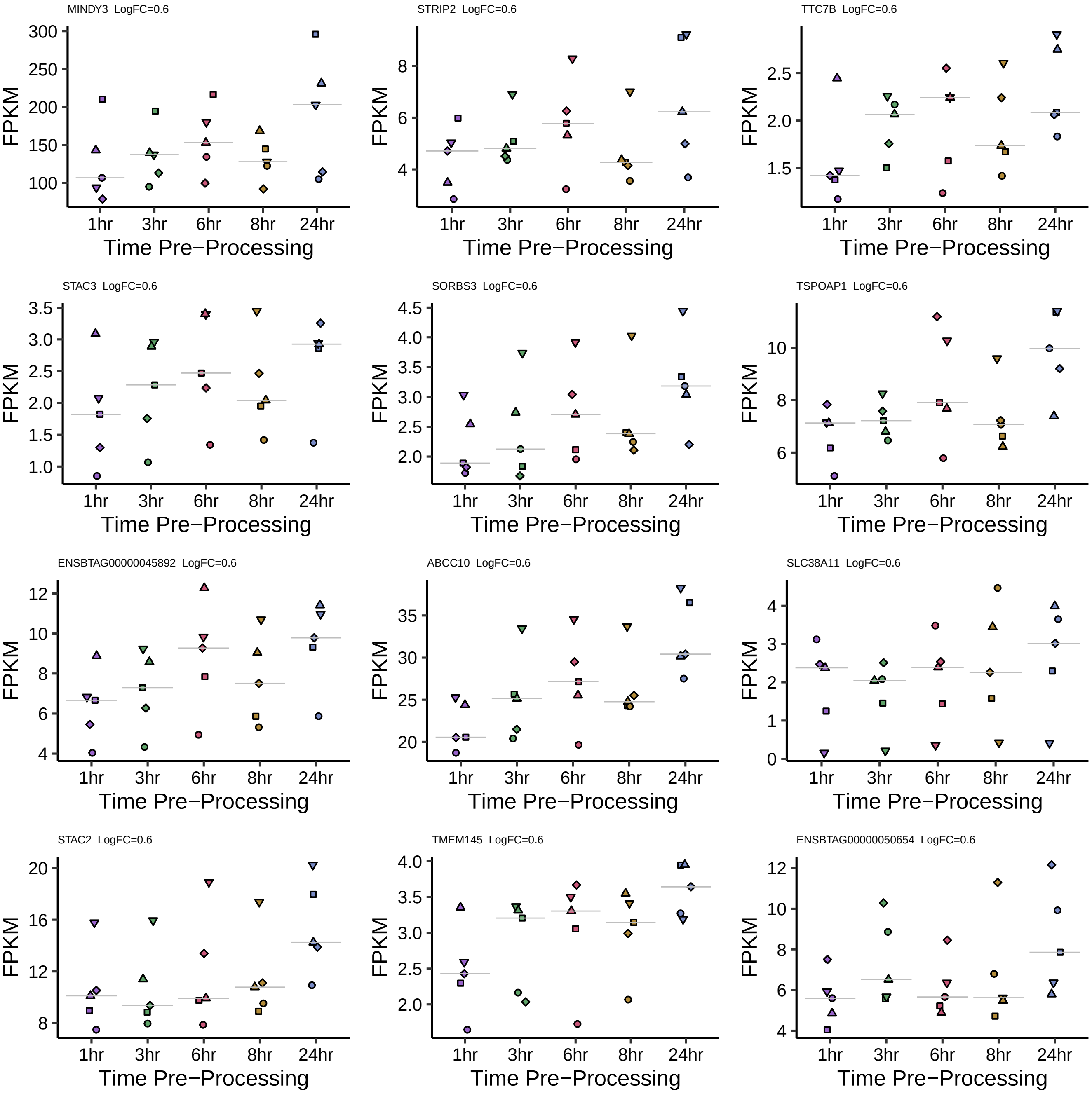
MINDY3 LogFC=0.6
STRIP2 LogFC=0.6
TTC7B LogFC=0.6
STAC3 LogFC=0.6
SORBS3 LogFC=0.6
TSPOAP1 LogFC=0.6
ENSBTAG00000045892 LogFC=0.6
ABCC10 LogFC=0.6
SLC38A11 LogFC=0.6
STAC2 LogFC=0.6
TMEM145 LogFC=0.6
ENSBTAG00000050654 LogFC=0.6
FPKM
1hr
3hr
6hr
8hr
24hr
Time Pre−Processing

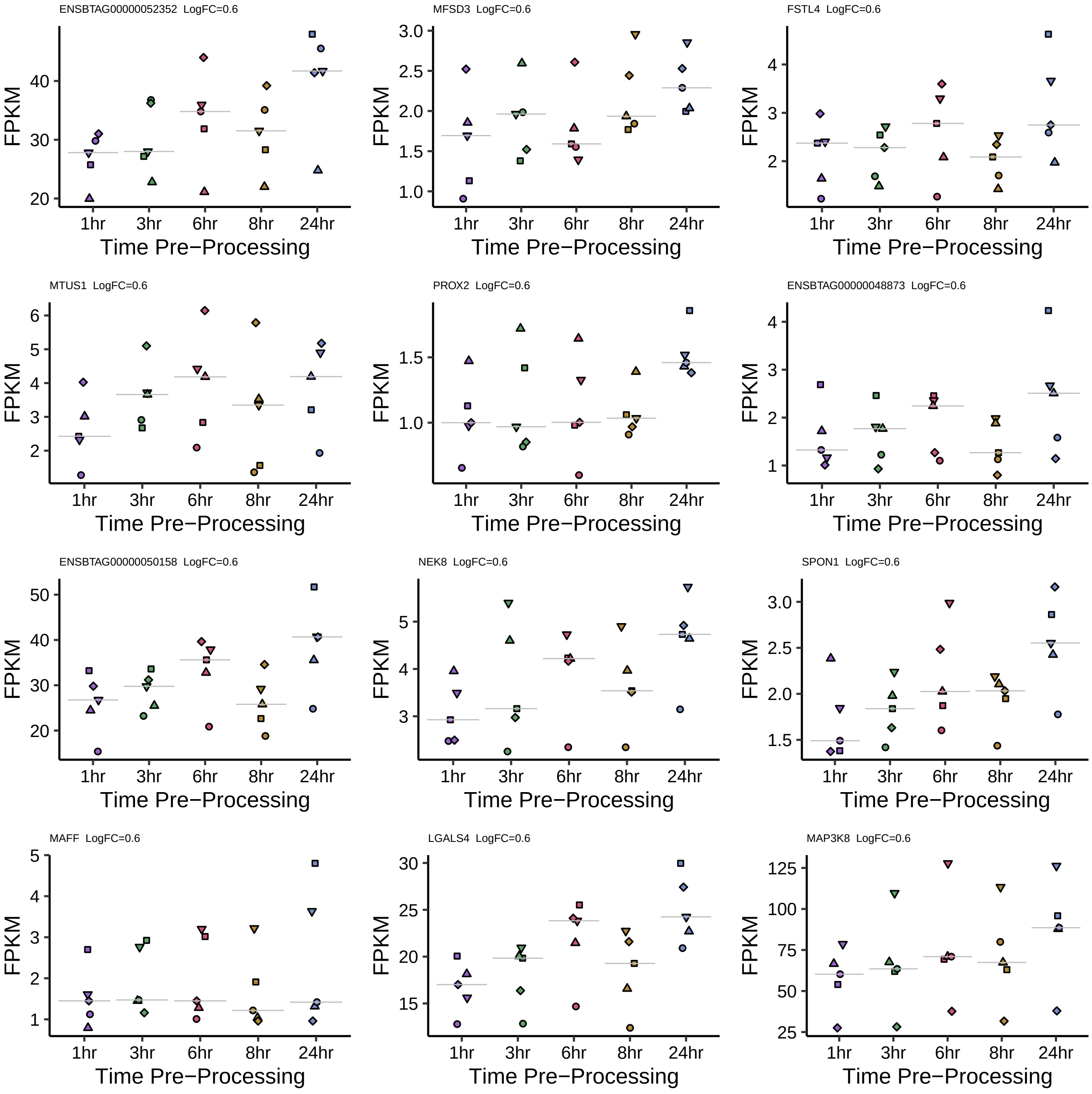

ENSBTAG00000052352 LogFC=0.6
MFSD3 LogFC=0.6
FSTL4 LogFC=0.6
MTUS1 LogFC=0.6
PROX2 LogFC=0.6
ENSBTAG00000048873 LogFC=0.6
ENSBTAG00000050158 LogFC=0.6
NEK8 LogFC=0.6
SPON1 LogFC=0.6
MAFF LogFC=0.6
LGALS4 LogFC=0.6
MAP3K8 LogFC=0.6
FPKM
1hr
3hr
6hr
8hr
24hr
Time Pre−Processing

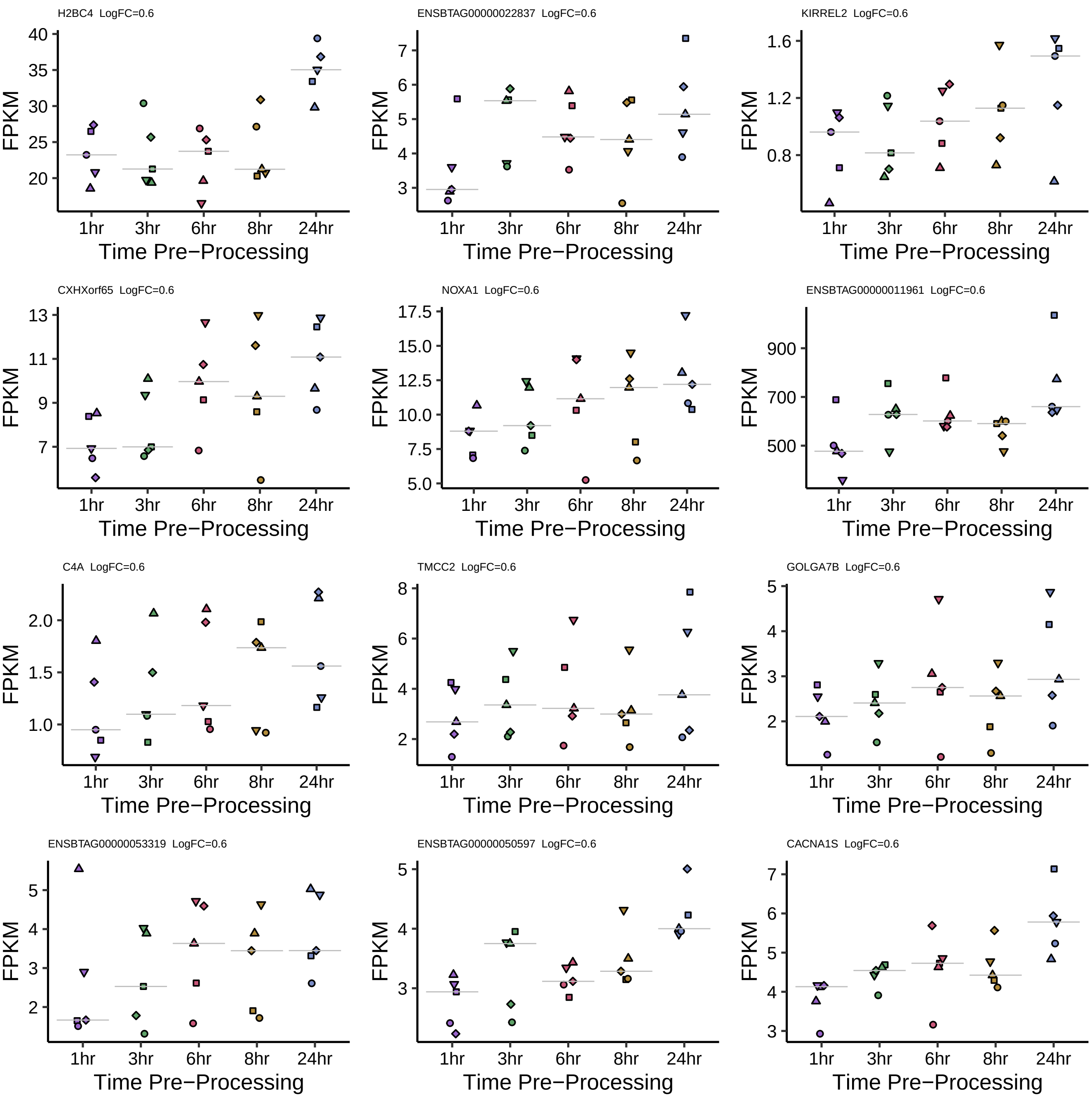

H2BC4 LogFC=0.6
ENSBTAG00000022837 LogFC=0.6
KIRREL2 LogFC=0.6
CXHXorf65 LogFC=0.6
NOXA1 LogFC=0.6
ENSBTAG00000011961 LogFC=0.6
C4A LogFC=0.6
TMCC2 LogFC=0.6
GOLGA7B LogFC=0.6
ENSBTAG00000053319 LogFC=0.6
ENSBTAG00000050597 LogFC=0.6
CACNA1S LogFC=0.6
FPKM
1hr
3hr
6hr
8hr
24hr
Time Pre−Processing

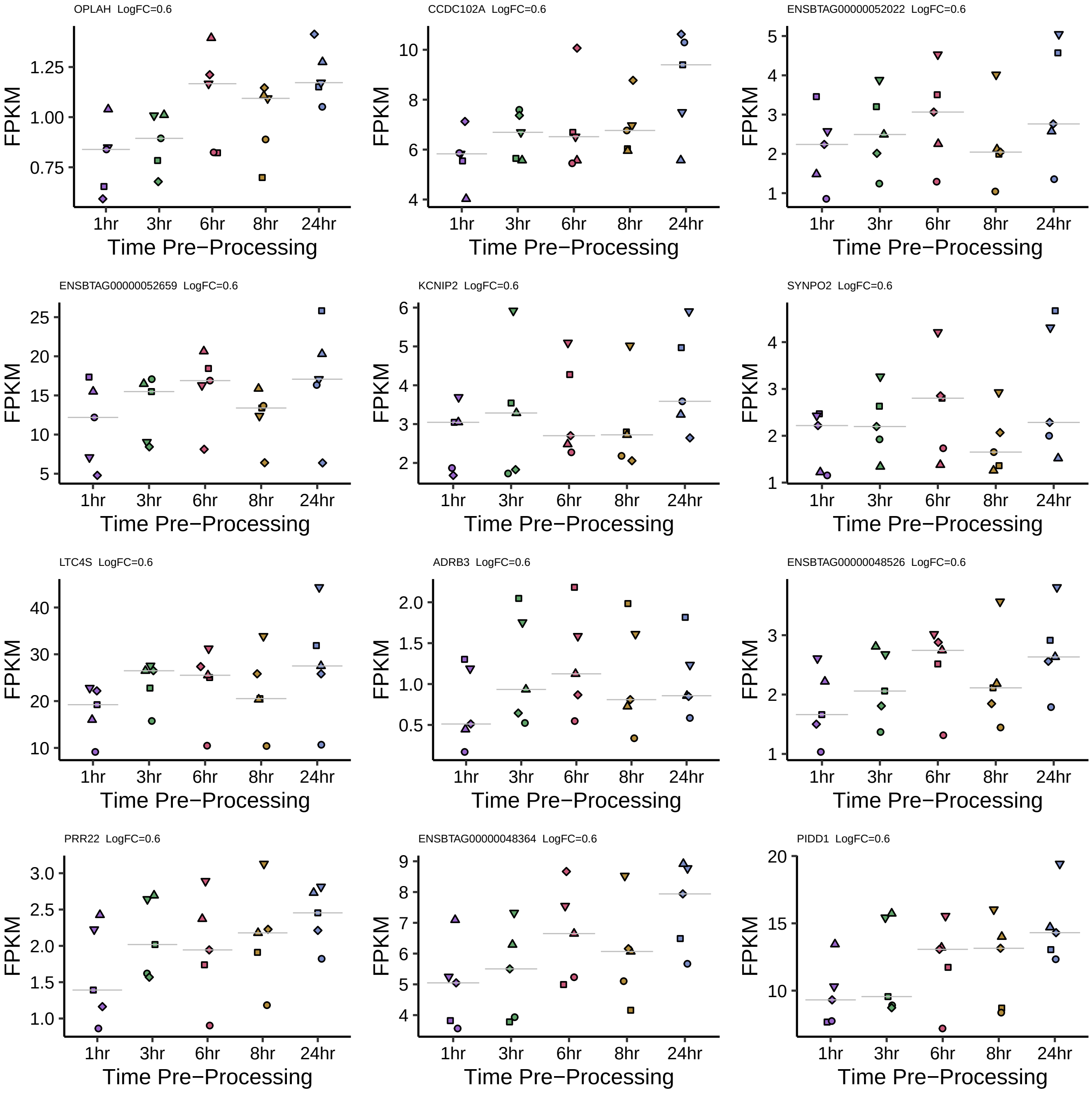
OPLAH LogFC=0.6
FPKM
1hr 3hr 6hr 8hr 24hr
Time Pre−Processing
CCDC102A LogFC=0.6
FPKM
1hr 3hr 6hr 8hr 24hr
Time Pre−Processing
ENSBTAG00000052022 LogFC=0.6
FPKM
1hr 3hr 6hr 8hr 24hr
Time Pre−Processing
ENSBTAG00000052659 LogFC=0.6
FPKM
1hr 3hr 6hr 8hr 24hr
Time Pre−Processing
KCNIP2 LogFC=0.6
FPKM
1hr 3hr 6hr 8hr 24hr
Time Pre−Processing
SYNPO2 LogFC=0.6
FPKM
1hr 3hr 6hr 8hr 24hr
Time Pre−Processing
LTC4S LogFC=0.6
FPKM
1hr 3hr 6hr 8hr 24hr
Time Pre−Processing
ADRB3 LogFC=0.6
FPKM
1hr 3hr 6hr 8hr 24hr
Time Pre−Processing
ENSBTAG00000048526 LogFC=0.6
FPKM
1hr 3hr 6hr 8hr 24hr
Time Pre−Processing
PRR22 LogFC=0.6
FPKM
1hr 3hr 6hr 8hr 24hr
Time Pre−Processing
ENSBTAG00000048364 LogFC=0.6
FPKM
1hr 3hr 6hr 8hr 24hr
Time Pre−Processing
PIDD1 LogFC=0.6
FPKM
1hr 3hr 6hr 8hr 24hr
Time Pre−Processing

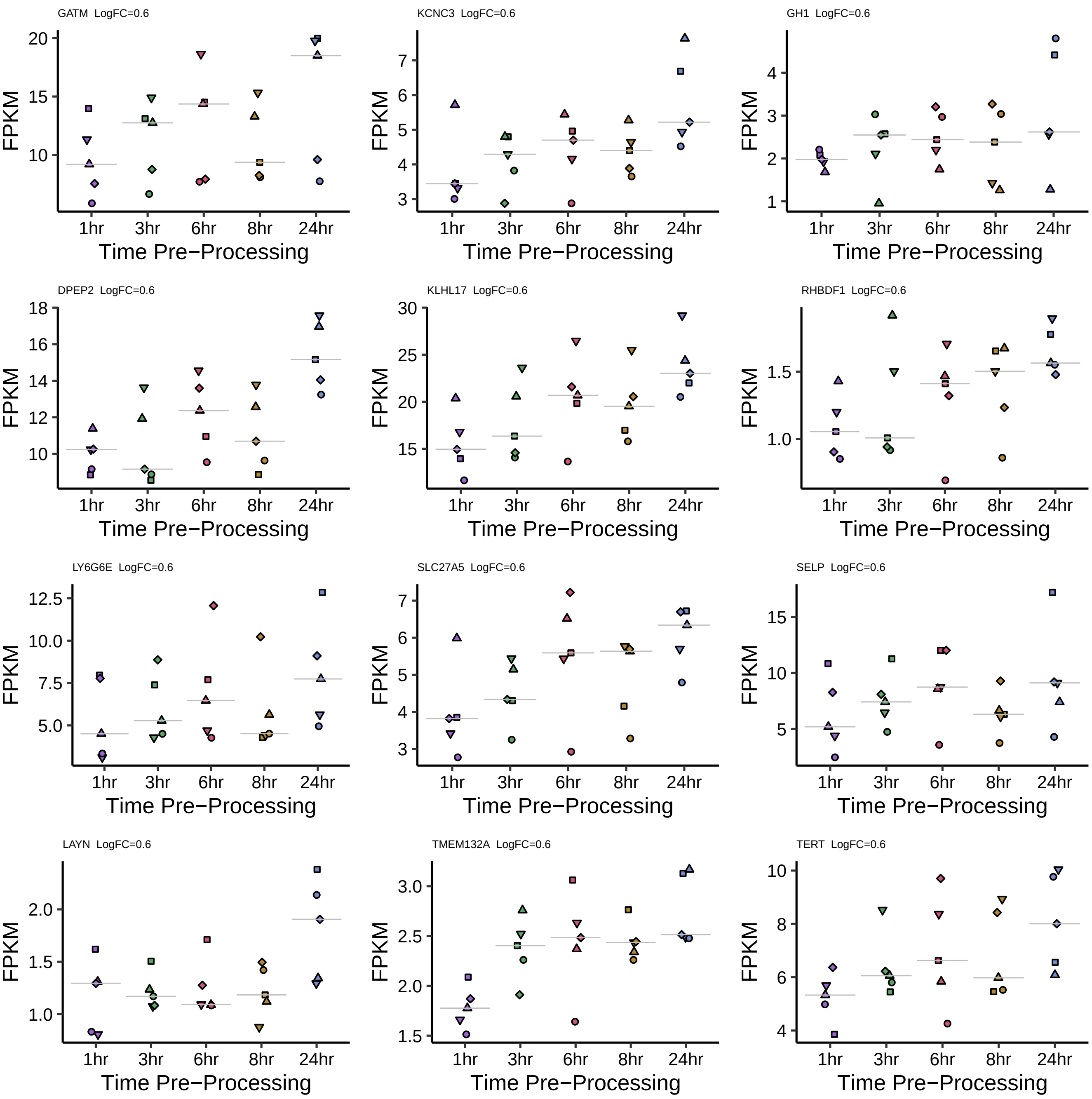

GATM LogFC=0.6
KCNC3 LogFC=0.6
GH1 LogFC=0.6
DPEP2 LogFC=0.6
KLHL17 LogFC=0.6
RHBDF1 LogFC=0.6
LY6G6E LogFC=0.6
SLC27A5 LogFC=0.6
SELP LogFC=0.6
LAYN LogFC=0.6
TMEM132A LogFC=0.6
TERT LogFC=0.6
FPKM
1hr 3hr 6hr 8hr 24hr
Time Pre−Processing

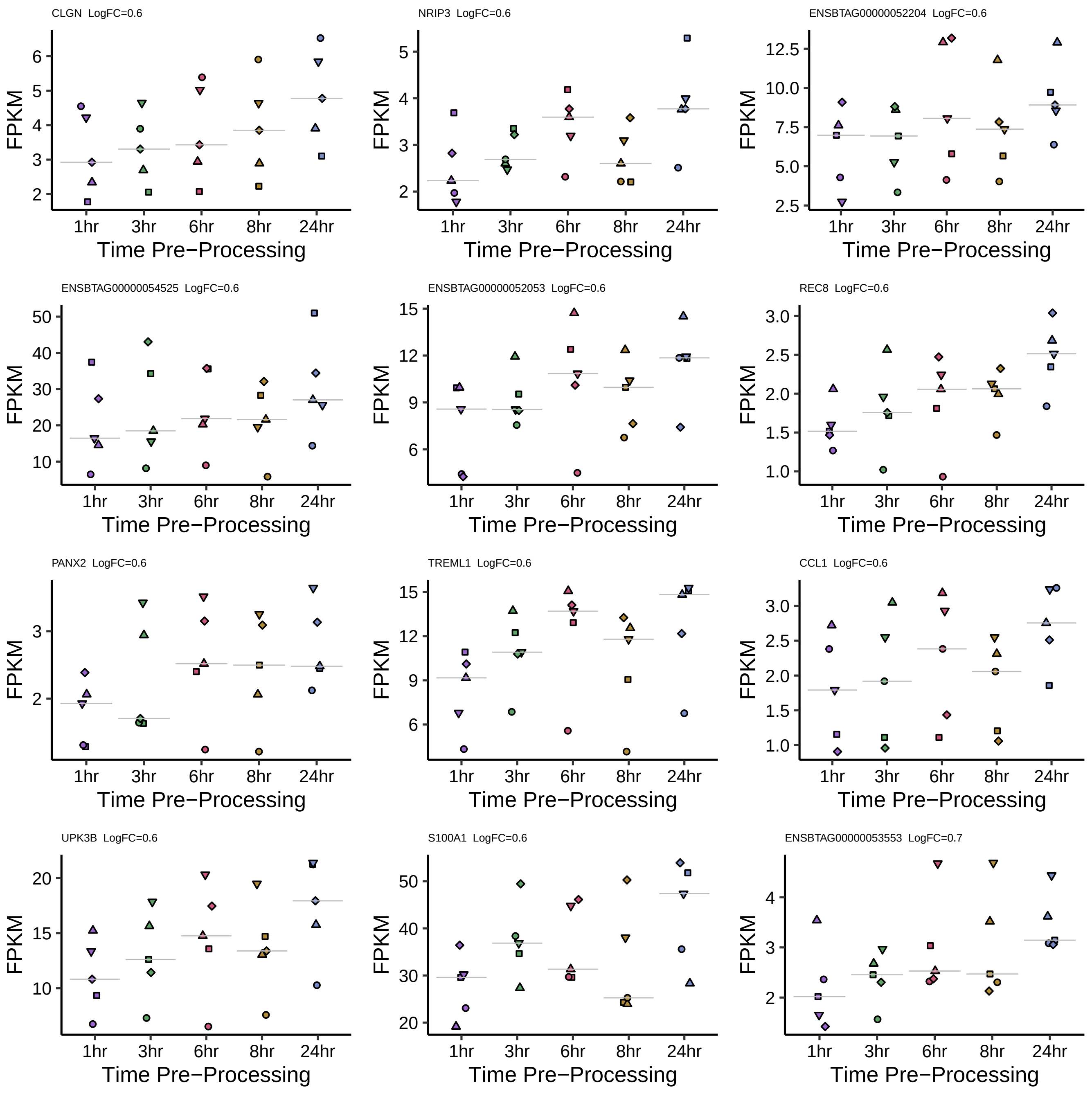
CLGN LogFC=0.6
NRIP3 LogFC=0.6
ENSBTAG00000052204 LogFC=0.6
ENSBTAG00000054525 LogFC=0.6
ENSBTAG00000052053 LogFC=0.6
REC8 LogFC=0.6
PANX2 LogFC=0.6
TREML1 LogFC=0.6
CCL1 LogFC=0.6
UPK3B LogFC=0.6
S100A1 LogFC=0.6
ENSBTAG00000053553 LogFC=0.7
FPKM
Time Pre−Processing
1hr
3hr
6hr
8hr
24hr

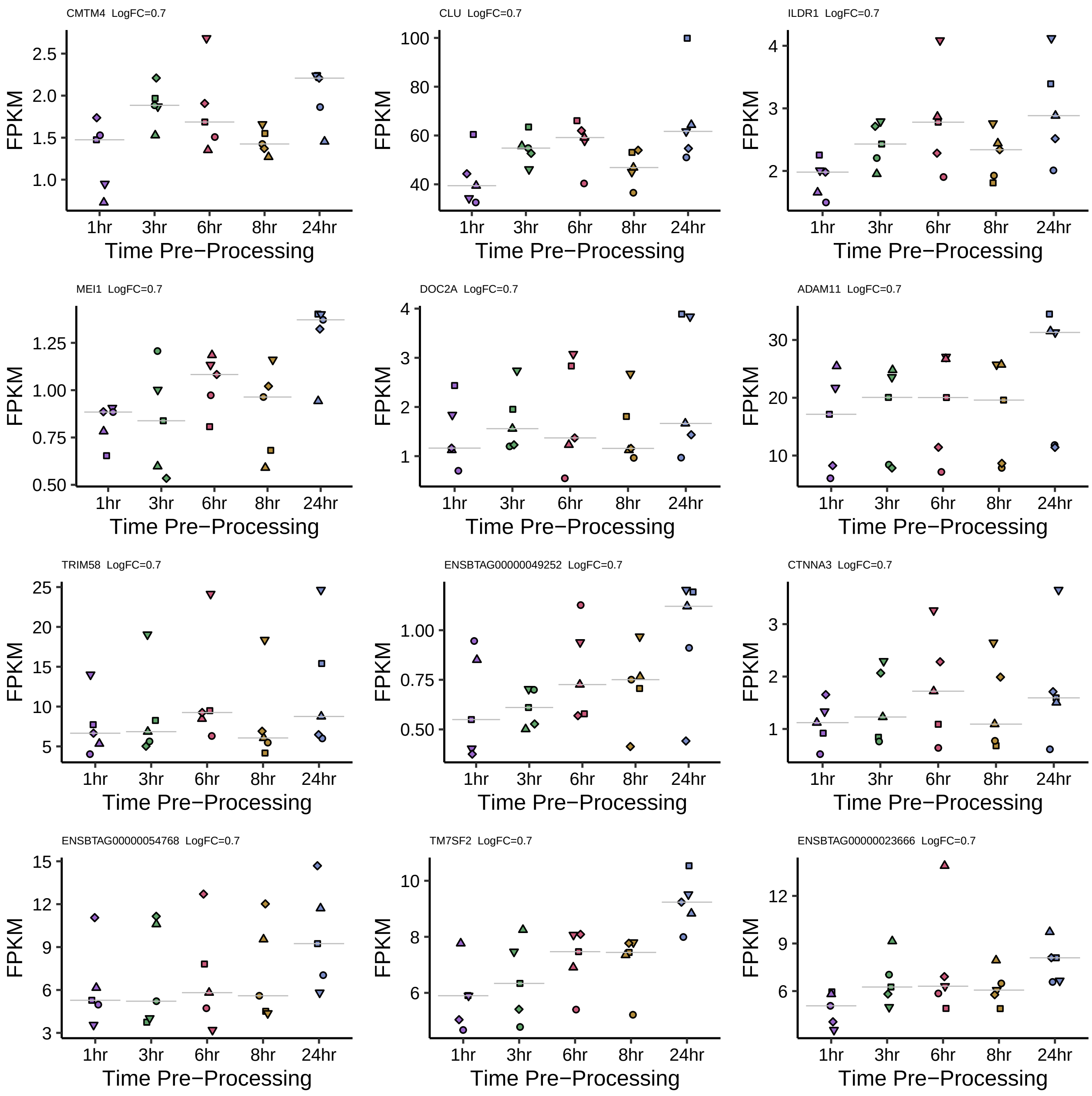

CMTM4 LogFC=0.7
CLU LogFC=0.7
ILDR1 LogFC=0.7
MEI1 LogFC=0.7
DOC2A LogFC=0.7
ADAM11 LogFC=0.7
TRIM58 LogFC=0.7
ENSBTAG00000049252 LogFC=0.7
CTNNA3 LogFC=0.7
ENSBTAG00000054768 LogFC=0.7
TM7SF2 LogFC=0.7
ENSBTAG00000023666 LogFC=0.7
FPKM
1hr
3hr
6hr
8hr
24hr
Time Pre−Processing

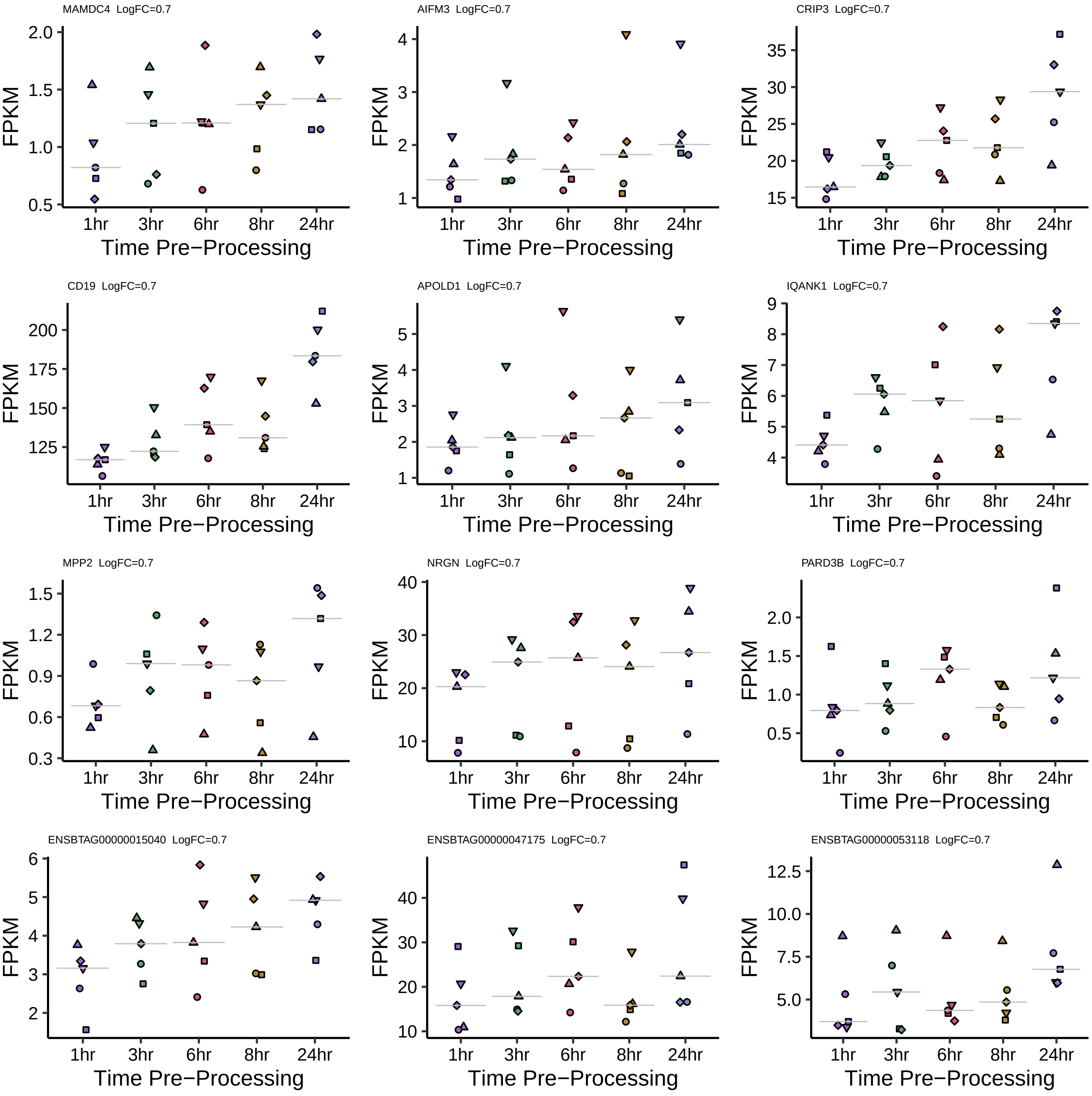
MAMDC4 LogFC=0.7
AIFM3 LogFC=0.7
CRIP3 LogFC=0.7
CD19 LogFC=0.7
APOLD1 LogFC=0.7
IQANK1 LogFC=0.7
MPP2 LogFC=0.7
NRGN LogFC=0.7
PARD3B LogFC=0.7
ENSBTAG00000015040 LogFC=0.7
ENSBTAG00000047175 LogFC=0.7
ENSBTAG00000053118 LogFC=0.7
FPKM
1hr 3hr 6hr 8hr 24hr
Time Pre−Processing

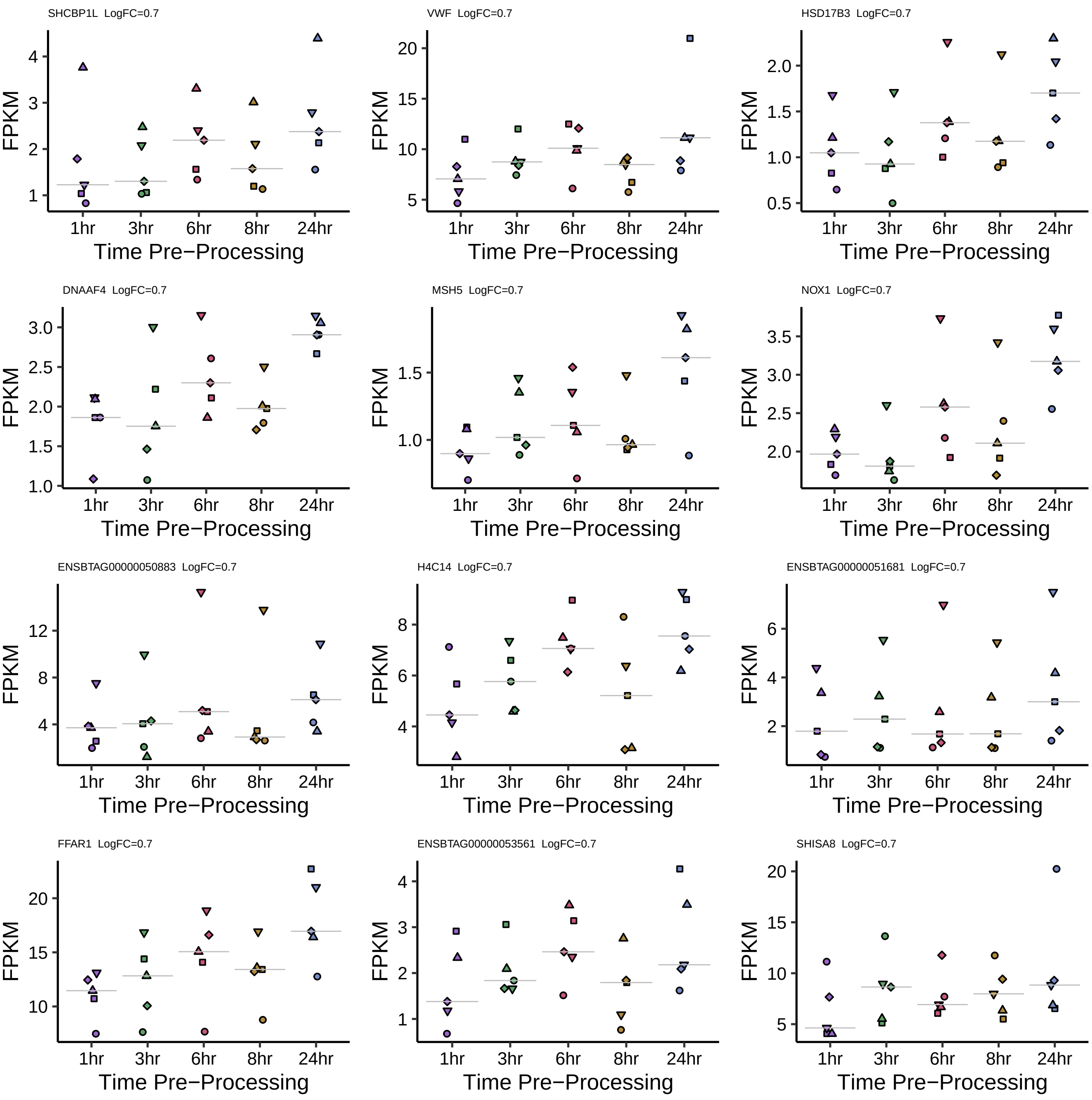
SHCBP1L LogFC=0.7
VWF LogFC=0.7
HSD17B3 LogFC=0.7
DNAAF4 LogFC=0.7
MSH5 LogFC=0.7
NOX1 LogFC=0.7
ENSBTAG00000050883 LogFC=0.7
H4C14 LogFC=0.7
ENSBTAG00000051681 LogFC=0.7
FFAR1 LogFC=0.7
ENSBTAG00000053561 LogFC=0.7
SHISA8 LogFC=0.7
FPKM
1hr
3hr
6hr
8hr
24hr
Time Pre−Processing

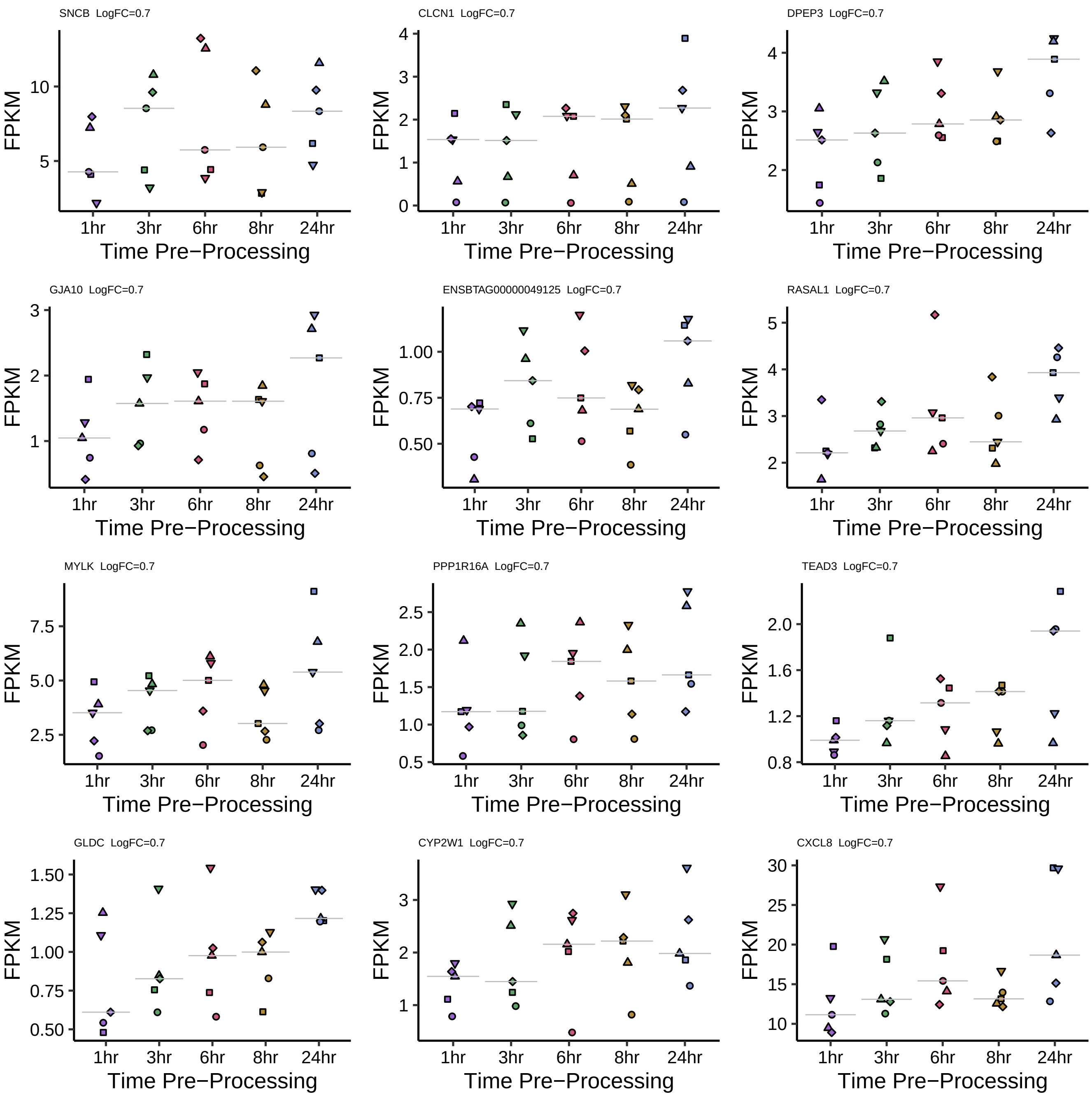

SNCB LogFC=0.7
CLCN1 LogFC=0.7
DPEP3 LogFC=0.7
GJA10 LogFC=0.7
ENSBTAG00000049125 LogFC=0.7
RASAL1 LogFC=0.7
MYLK LogFC=0.7
PPP1R16A LogFC=0.7
TEAD3 LogFC=0.7
GLDC LogFC=0.7
CYP2W1 LogFC=0.7
CXCL8 LogFC=0.7
FPKM
1hr
3hr
6hr
8hr
24hr
Time Pre−Processing

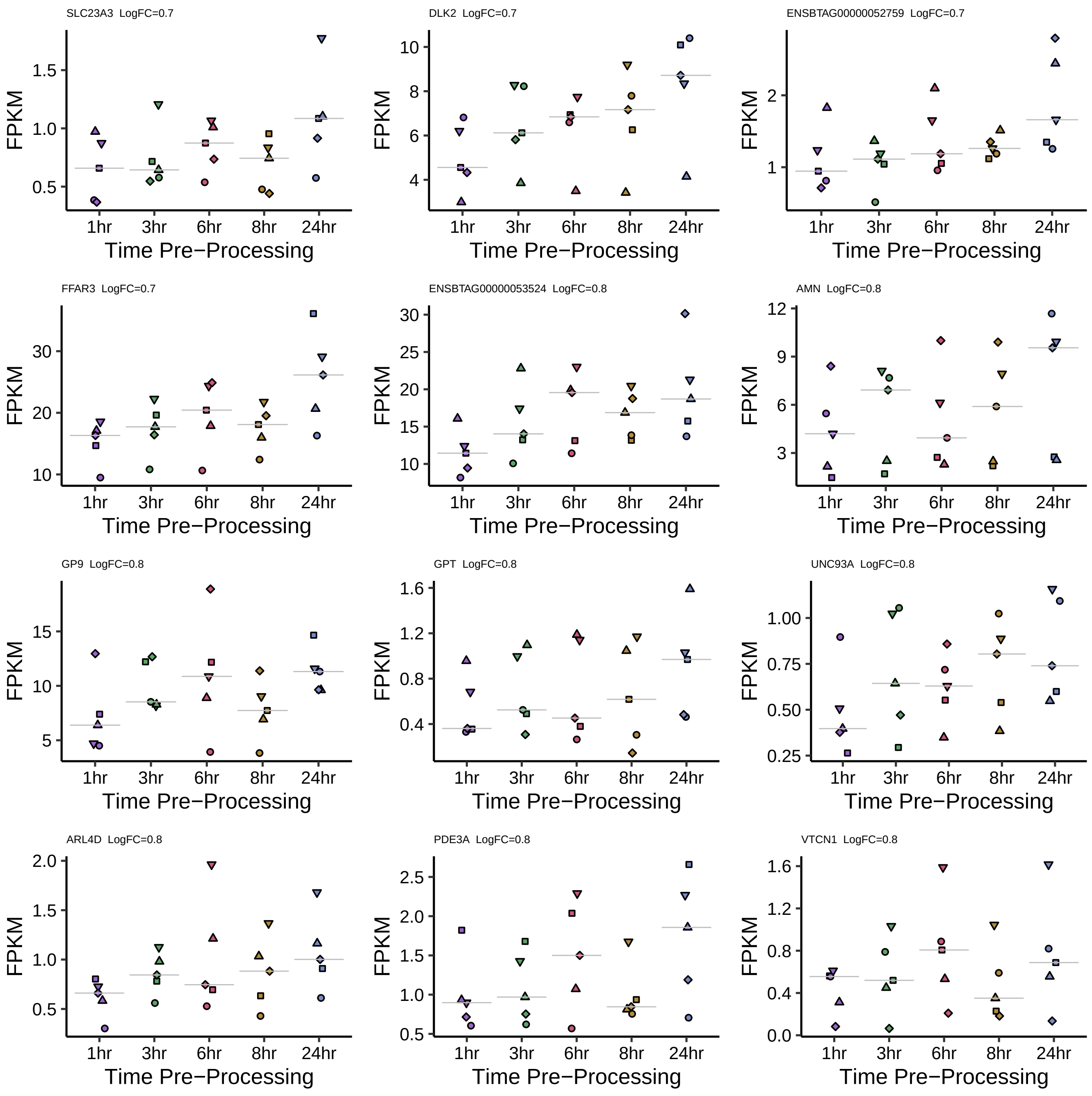

SLC23A3 LogFC=0.7
DLK2 LogFC=0.7
ENSBTAG00000052759 LogFC=0.7
FFAR3 LogFC=0.7
ENSBTAG00000053524 LogFC=0.8
AMN LogFC=0.8
GP9 LogFC=0.8
GPT LogFC=0.8
UNC93A LogFC=0.8
ARL4D LogFC=0.8
PDE3A LogFC=0.8
VTCN1 LogFC=0.8
FPKM
1hr
3hr
6hr
8hr
24hr
Time Pre−Processing

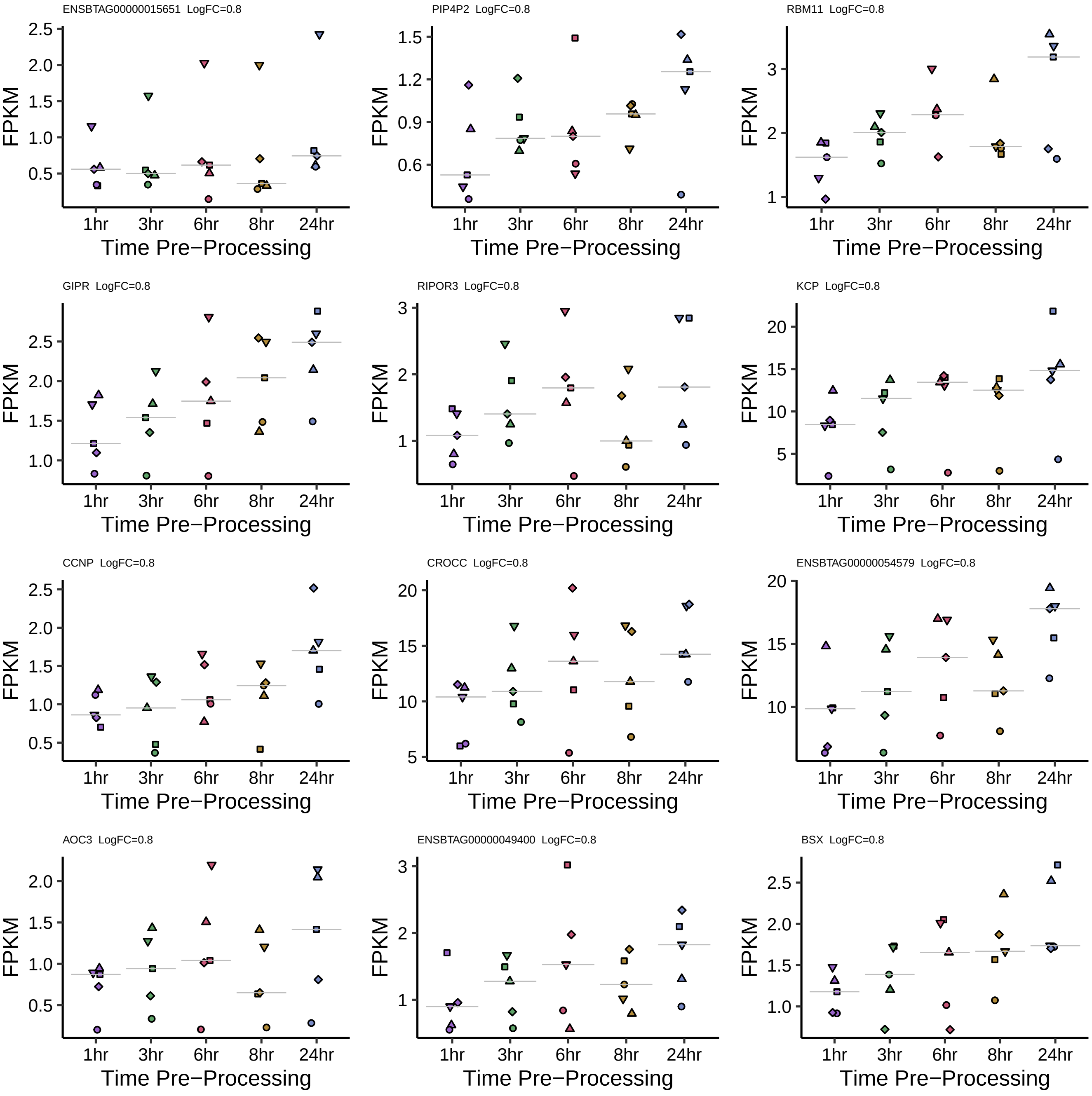
ENSBTAG00000015651 LogFC=0.8
PIP4P2 LogFC=0.8
RBM11 LogFC=0.8
GIPR LogFC=0.8
RIPOR3 LogFC=0.8
KCP LogFC=0.8
CCNP LogFC=0.8
CROCC LogFC=0.8
ENSBTAG00000054579 LogFC=0.8
AOC3 LogFC=0.8
ENSBTAG00000049400 LogFC=0.8
BSX LogFC=0.8
FPKM
1hr
3hr
6hr
8hr
24hr
Time Pre−Processing

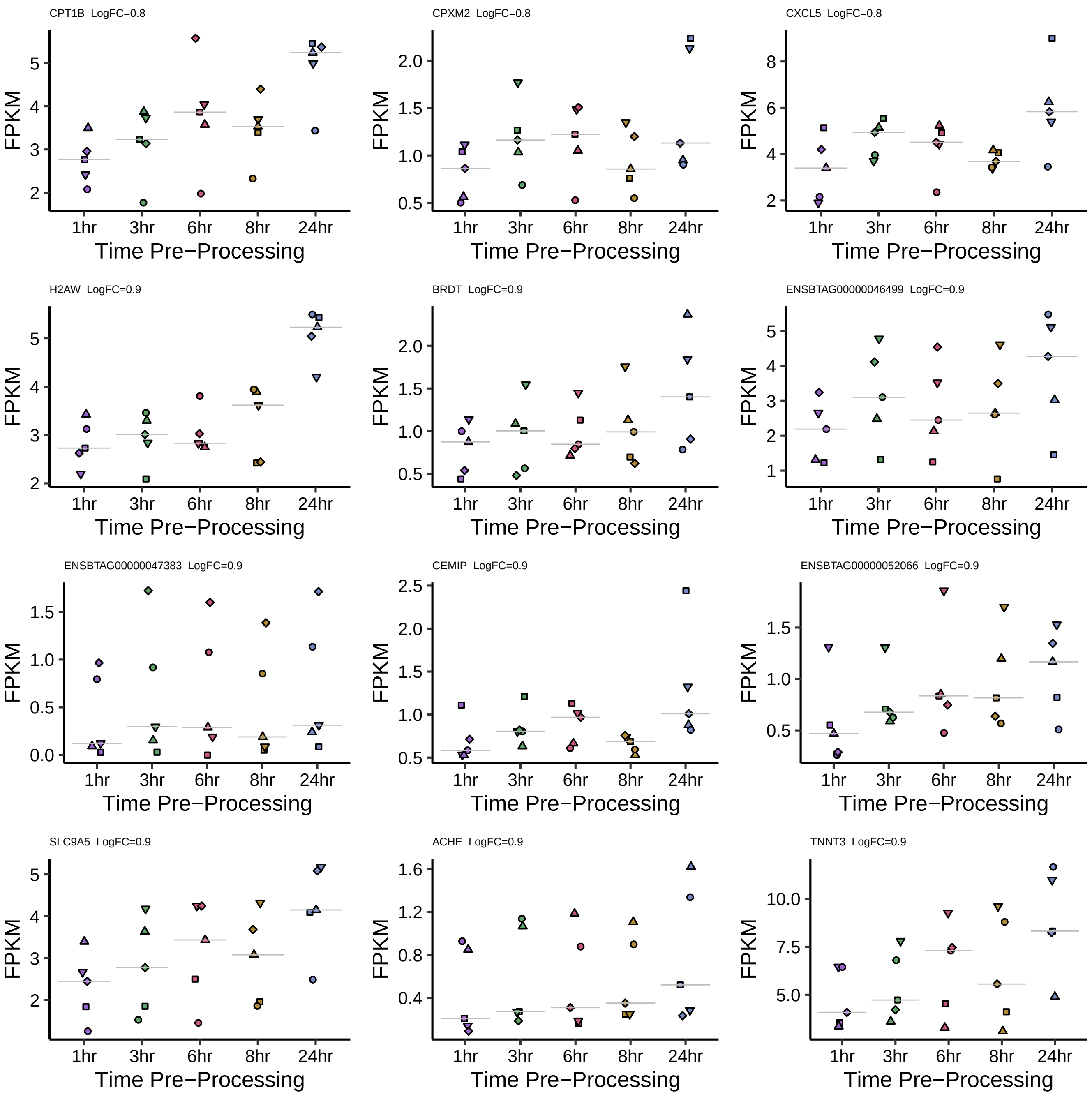

CPT1B LogFC=0.8
CPXM2 LogFC=0.8
CXCL5 LogFC=0.8
H2AW LogFC=0.9
BRDT LogFC=0.9
ENSBTAG00000046499 LogFC=0.9
ENSBTAG00000047383 LogFC=0.9
CEMIP LogFC=0.9
ENSBTAG00000052066 LogFC=0.9
SLC9A5 LogFC=0.9
ACHE LogFC=0.9
TNNT3 LogFC=0.9
FPKM
1hr
3hr
6hr
8hr
24hr
Time Pre−Processing

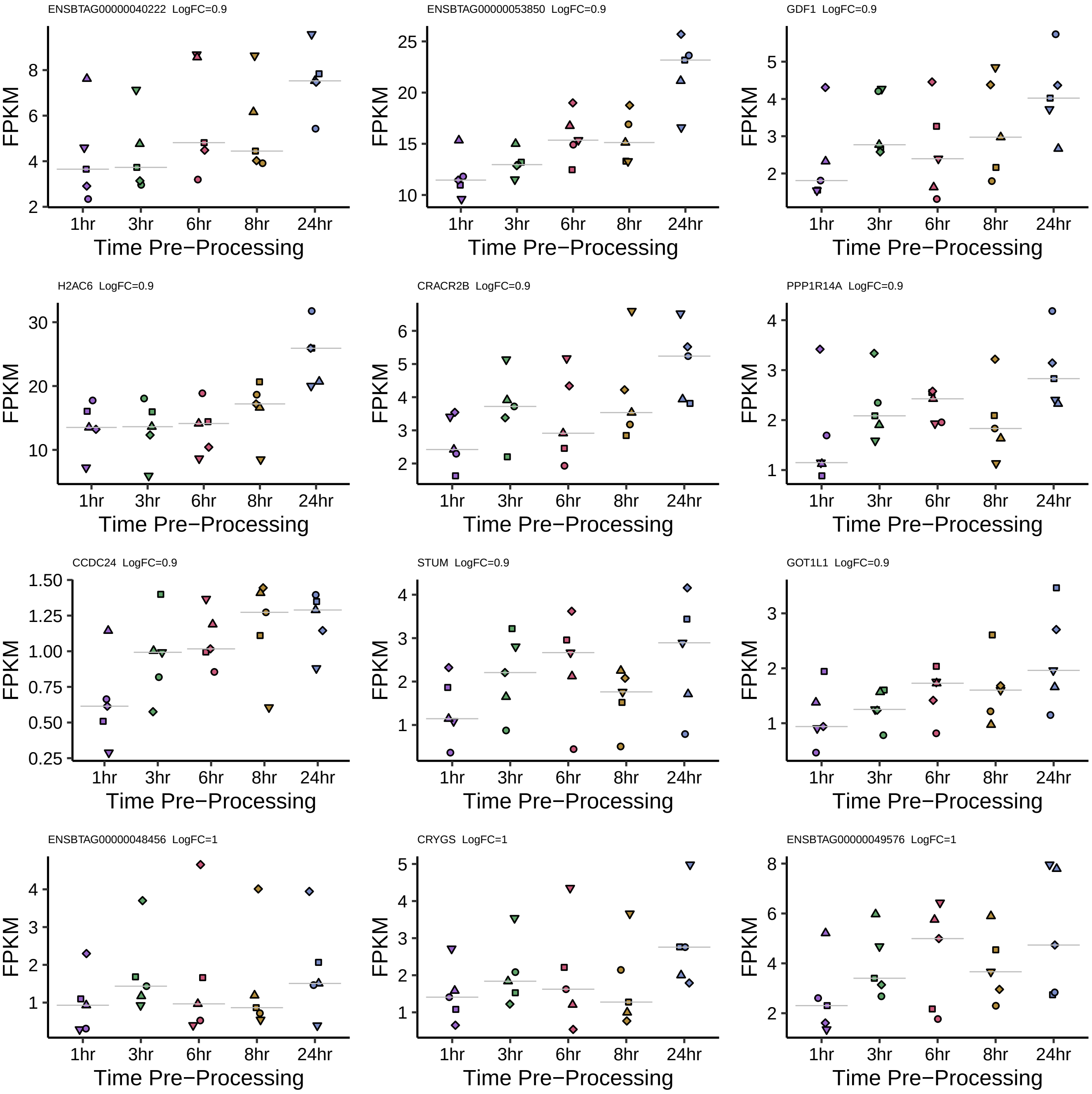

ENSBTAG00000040222 LogFC=0.9
ENSBTAG00000053850 LogFC=0.9
GDF1 LogFC=0.9
H2AC6 LogFC=0.9
CRACR2B LogFC=0.9
PPP1R14A LogFC=0.9
CCDC24 LogFC=0.9
STUM LogFC=0.9
GOT1L1 LogFC=0.9
ENSBTAG00000048456 LogFC=1
CRYGS LogFC=1
ENSBTAG00000049576 LogFC=1
FPKM
1hr
3hr
6hr
8hr
24hr
Time Pre−Processing

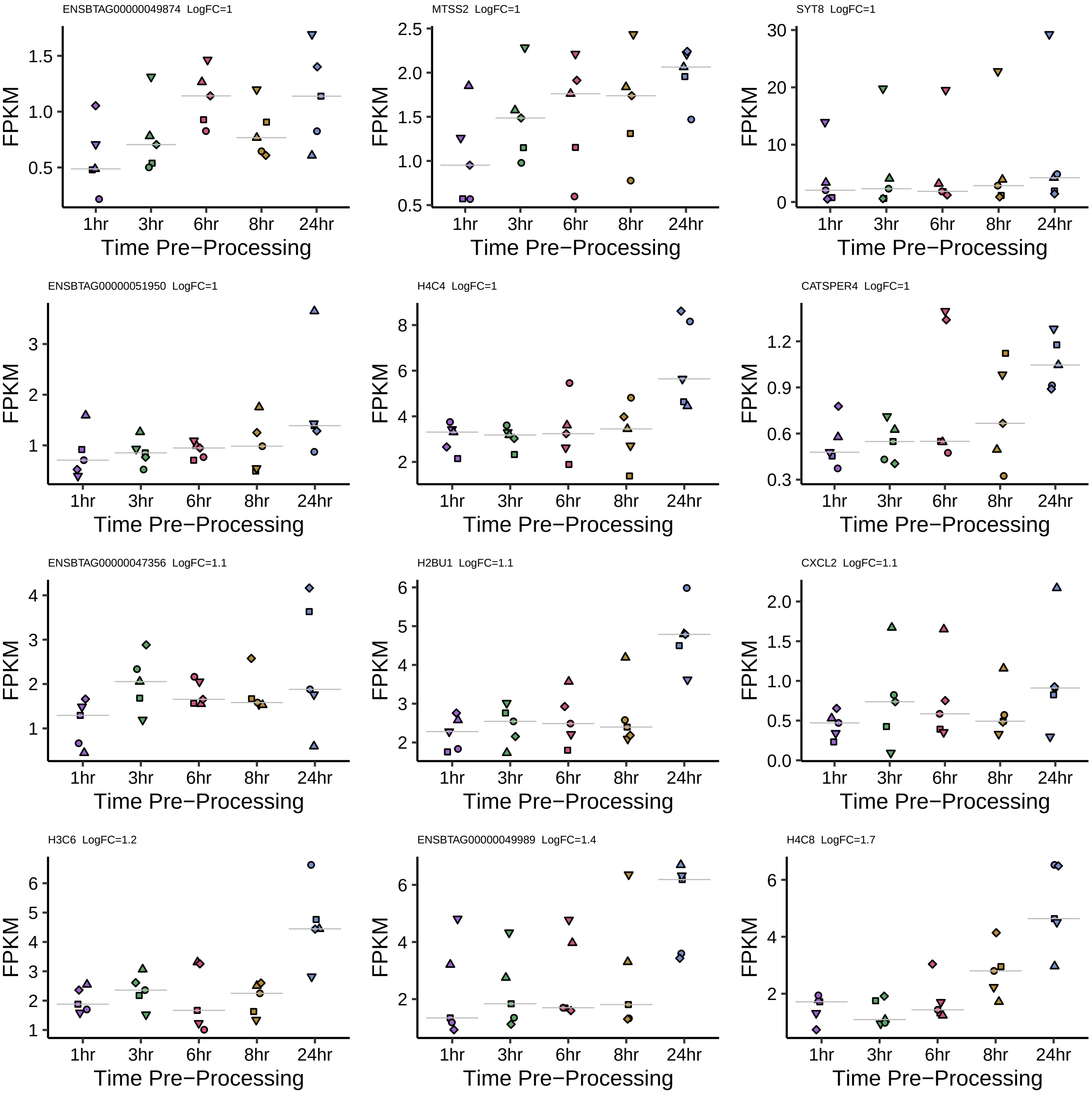
ENSBTAG00000049874 LogFC=1
MTSS2 LogFC=1
SYT8 LogFC=1
ENSBTAG00000051950 LogFC=1
H4C4 LogFC=1
CATSPER4 LogFC=1
ENSBTAG00000047356 LogFC=1.1
H2BU1 LogFC=1.1
CXCL2 LogFC=1.1
H3C6 LogFC=1.2
ENSBTAG00000049989 LogFC=1.4
H4C8 LogFC=1.7
FPKM
1hr
3hr
6hr
8hr
24hr
Time Pre−Processing

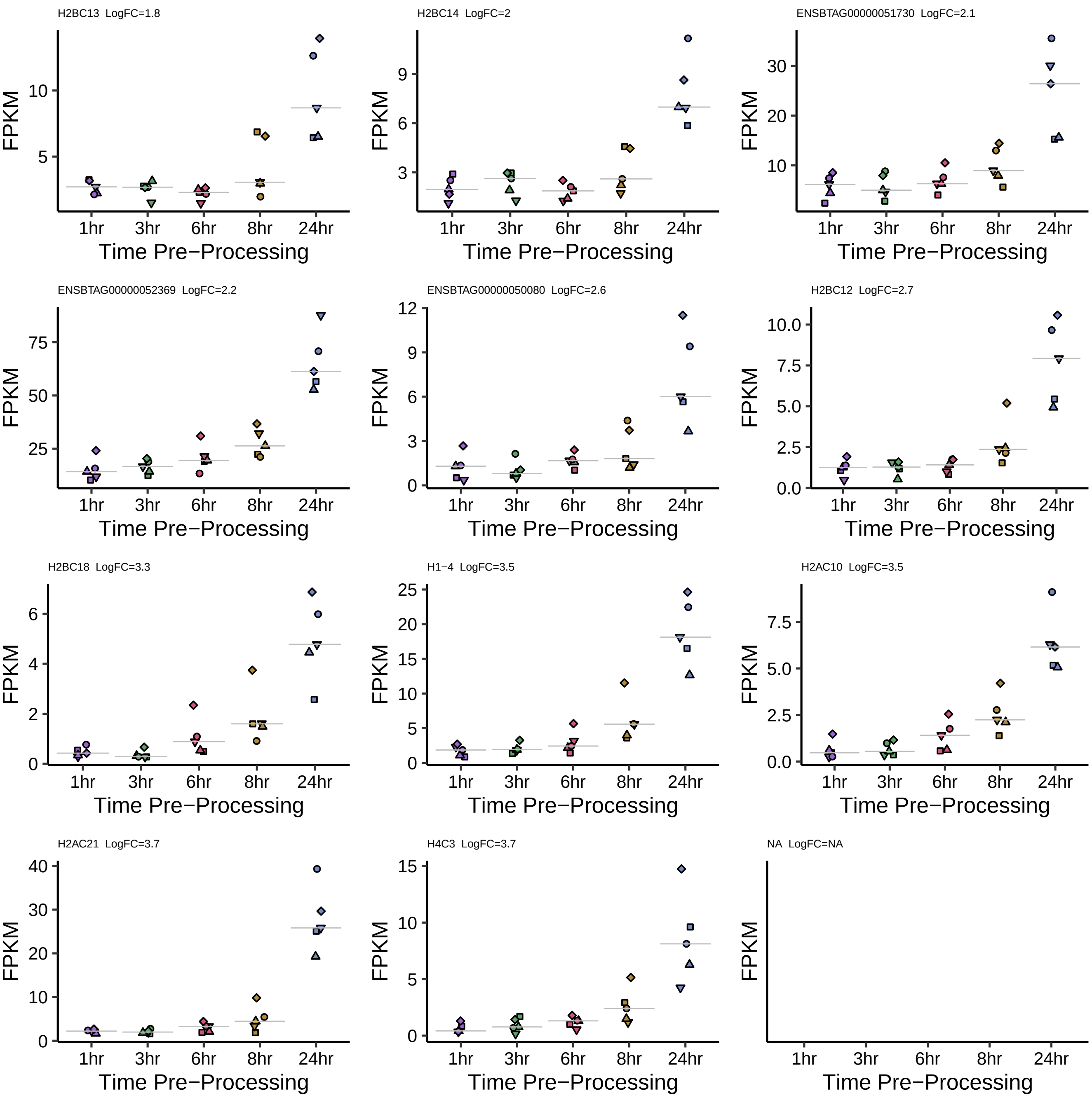
H2BC13 LogFC=1.8
H2BC14 LogFC=2
ENSBTAG00000051730 LogFC=2.1
ENSBTAG00000052369 LogFC=2.2
ENSBTAG00000050080 LogFC=2.6
H2BC12 LogFC=2.7
H2BC18 LogFC=3.3
H1−4 LogFC=3.5
H2AC10 LogFC=3.5
H2AC21 LogFC=3.7
H4C3 LogFC=3.7
NA LogFC=NA
FPKM
1hr
3hr
6hr
8hr
24hr
Time Pre−Processing

Supplementary Fig. S5. Charts of transcript abundance (fragment per kilobase per million reads, FPKM) for different times of refrigeration (4°C) prior to processing and cryopreservation of PWBCs. Animals are indicated by shapes across charts.

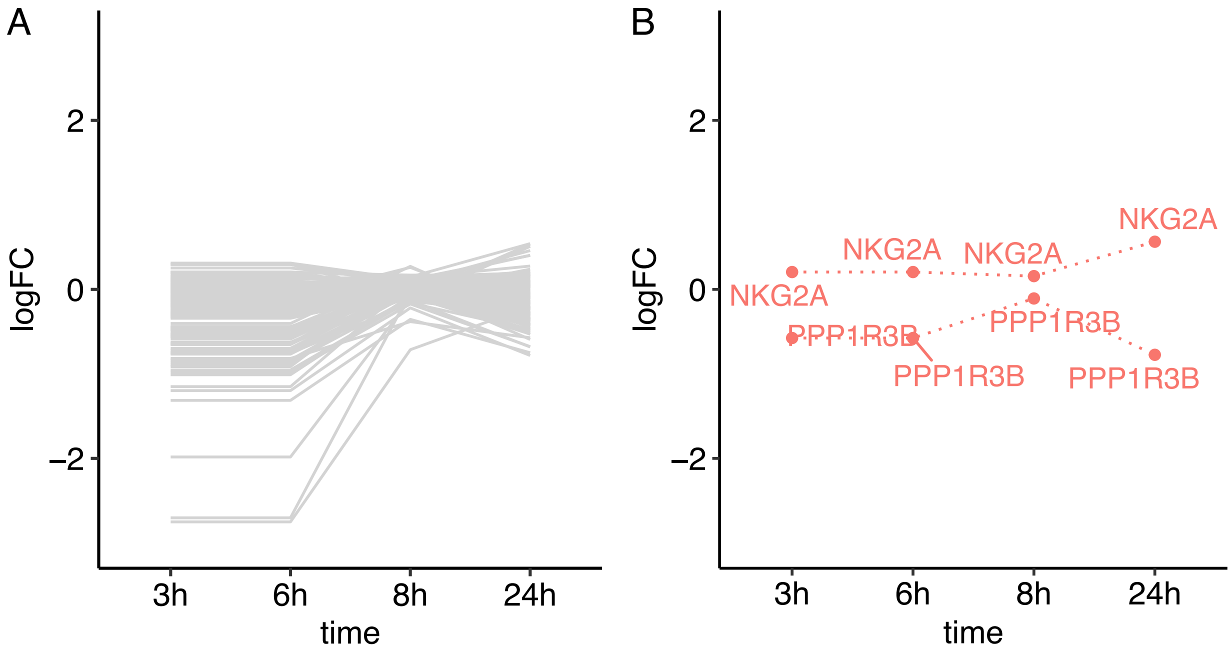

Supplementary Fig. S6. Differential transcript abundance for genes previously detected as potential biomarkers of heifer fertility. **A**. Genes with no significant variation of transcript abundance following three, six, eight or 24 hours of refrigeration post collection versus one hour within collection. **B**. Genes with significantly differential transcript abundance following 24 hours of refrigeration post collection versus one hour within collection.
