# Supplementary material for: Delayed processing of blood samples impairs the accuracy of mRNA-based biomarkers
Source: Sci Rep. 2022 May 17;12:8196. doi: 10.1038/s41598-022-12178-5 (PMC9113984; doi:10.1038/s41598-022-12178-5)
Supplement: Supplementary file 8 — Supplementary Information 8. [file 41598_2022_12178_MOESM8_ESM.pdf]

Supplementary table S6. List of reagents and kits with their catalog number.

Kit/Reagent	Company	Catalog Number
Agilent RNA 6000 Pico Kit	Agilent	5067-1513
AMPure XP	Beckman Coulter	A63881
Chloroform	Thermo Scientific	AAJ67241AP
Ethanol	Fisher BioReagents	BP2818500
Isopropanol	Thermo Scientific	AC184130010
Phasemaker Tubes	Invitrogen	A33248
Qubit RNA High Sensitivity Kit	Invitrogen	Q32852
TRIzol Reagent	Invitrogen	15596018
TruSeq Stranded mRNA Library Prep	Illumina	20020595
